# PVN^CRF^ Neurons Regulate Migraine‐Like Allodynia by Activating CRFR2 on Spinal Trigeminal Caudalis Glutamatergic Neurons

**DOI:** 10.1002/advs.202520530

**Published:** 2026-02-28

**Authors:** Jiang Bian, Xiao‐Long Wang, Li Yin, Wei Wei, Xue Li, Yao Ge, Zhao‐Xia Xiang, Na Tian, Li‐Juan Chen, Ming‐Wei Ma, Xia Zhang, Xu Jing, Mei‐Yun Wang, Xu‐Feng Xu

**Affiliations:** ^1^ Neuropsychiatry Research Institute, Basic School of Medicine The Affiliated Hospital of Qingdao University Qingdao University Qingdao China; ^2^ Department of Anesthesiology Panzhihua Central Hospital Panzhihua China; ^3^ Department of Breast Surgery General Surgery Qilu Hospital of Shandong University Jinan Shandong China; ^4^ Department of Radiology Henan Provincial People's Hospital & State Key Laboratory of Metabolic Dysregulation & Prevention and Treatment of Esophageal Cancer Zhengzhou University Zhengzhou Henan China; ^5^ Department of Neurology Henan Provincial People's Hospital Zhengzhou University Zhengzhou Henan China; ^6^ Department of Microbiology, Tumor and Cell Biology Karolinska Institute Stockholm Sweden; ^7^ Breast Disease Center The First Affiliated Hospital, Sun Yat‐sen University Guangzhou China

**Keywords:** chronic migraine, corticotropin‐releasing factor, the hypothalamic paraventricular nucleus, the spinal trigeminal nucleus caudalis

## Abstract

Migraine is a prevalent and debilitating neurological disorder with poorly understood neural mechanisms. Here, we characterize a hypothalamic‐trigeminal pathway involved in the regulation of migraine‐like allodynia. Using a combination of monosynaptic circuit, fiber photometry‐based calcium imaging, and behavioral assays in a nitroglycerin (NTG)‐induced murine model, we observed hyperactivity in corticotropin‐releasing factor (CRF)‐expressing neurons within the hypothalamic paraventricular nucleus (PVN). Activation or inhibition of PVN^CRF^ neurons mimicked or blocked migraine‐like allodynia, respectively. These PVN^CRF^ neurons modulated migraine‐like allodynia by exciting glutamatergic neurons in the spinal trigeminal nucleus caudalis (SP5C). Furthermore, employing a CRF neurotransmitter fluorescent sensor, neuropharmacology, and electrophysiological recordings, we revealed that PVN^CRF^ neurons excessively release CRF neuropeptides onto SP5C^Glu^ neurons during migraine‐like conditions. This led to hyperactivation of corticotropin‐releasing factor receptor type 2 (CRFR2), but not type 1 (CRFR1), resulting in hyperalgesia. Blockade of CRFR2 within the SP5C significantly alleviated migraine‐like allodynia. Complementing these findings, clinical functional magnetic resonance imaging (fMRI) of migraine patients indicated structural and functional alterations in PVN and SP5C regions associated with this pathway. Collectively, our results uncover a previously unappreciated PVN^CRF^‐SP5C^Glu^ pathway in migraine‐like allodynia, providing novel insights into the neurobiology of migraine and identifying potential therapeutic targets.

## Graphical abstract

Paraventricular CRF neurons in the hypothalamus project to glutamatergic neurons of the spinal trigeminal caudal nucleus, enhancing neuronal activation and CRFR2 signaling. This pathway increases CRF release and excitatory drive, promoting central sensitization and migraine‐related behaviors. Targeting the PVN–SP5C circuit provides a potential strategy for attenuating migraine sensitization. The scheme highlights a therapeutic entry point.

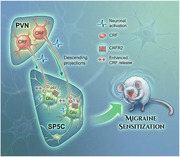

## 1 Introduction

Migraine is a prevalent and debilitating neurological disorder affecting approximately 14.4% of the global population, characterized by idiopathic unilateral throbbing headache often accompanied by sensory hypersensitivity, nausea, and vomiting [1]. Recognized by the World Health Organization as the third most common disorder and the second most disabling neurological condition, its pathophysiological mechanisms remain incompletely understood [2]. Evidence suggests cortical and subcortical brain regions collaboratively initiate and sustain migraine attacks [3, 4, 5]. Chronic migraine patients exhibit abnormalities in multiple regions of the central nervous system (CNS), including the hypothalamus [6], insula [7], prefrontal cortex [8] and trigeminocervical complex (TCC) [9]; however, the neuromodulatory basis of chronic migraine remains elusive.

The hypothalamic paraventricular nucleus (PVN) integrates interoceptive and exteroceptive information to regulate survival‐critical physiological processes and behaviors [10]. It plays a pivotal role in pain modulation through diverse neuropeptide‐producing neuronal populations. These neurons synthesize neuropeptides such as oxytocin (OXT), vasopressin, and corticotropin‐releasing factor (CRF), coordinating nociceptive processing [11, 12, 13]. Recent studies utilizing in vivo electrophysiological recordings have revealed that PVN electrical stimulation can inhibit nociceptive activity in wide dynamic range (WDR) neurons within the TCC region. Furthermore, local administration of an OXT receptor antagonist in the TCC partially reverses the inhibitory effect of PVN electrical stimulation on WDR neurons, thereby uncovering the role of the PVN oxytocinergic pathway in modulating trigeminal nociception [14]. Nonetheless, the mechanisms by which the PVN regulates migraine remain unclear. Critically, the precise functions and pathways mediated by PVN^CRF^ neurons—another crucial population of nociceptive modulators within the PVN—in migraine pathogenesis are still largely unknown.

In this study, we observed that PVN^CRF^ neurons were significantly activated under the nitroglycerin (NTG)‐induced migraine‐like condition. Utilizing a multidisciplinary approach, including fiber photometry‐based calcium imaging, chemogenetic manipulations, electrophysiological recordings, RNAscope in situ hybridization, and neuropharmacological interventions, we investigated the functional roles, neural circuitry, and receptor mechanisms of PVN^CRF^ neurons in the NTG‐induced mouse migraine model. To corroborate preclinical findings, we analyzed cranial fMRI data from migraine patients to identify potential structural and functional alterations in hypothalamic and brainstem regions. Integration of these clinical and experimental datasets reveals a previously unrecognized pro‐nociceptive role of PVN^CRF^ neurons in migraine, providing novel insights into the neurophysiological link between hypothalamic regulation and migraine pathophysiology.

## 2 Results

### 2.1 PVN^CRF^ Neurons Were Robustly Activated in an NTG‐Induced Chronic Migraine Model

**FIGURE 1:**
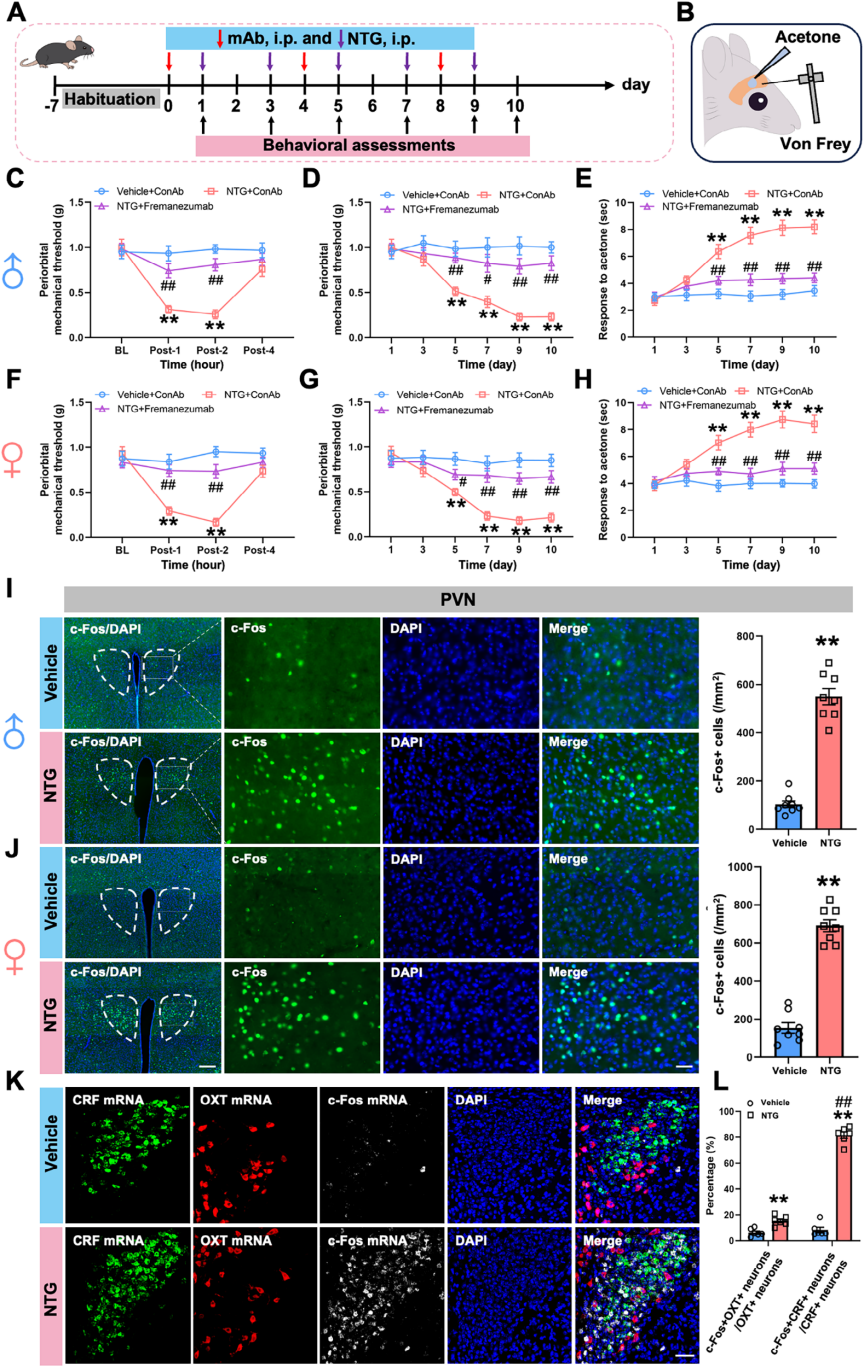
NTG injections elicit migraine‐like allodynia and induce PVN activation. (A). Experimental schedule for repeated NTG injections, CGRP antibody administrations and behavioral tests. (B) Schematic diagram of Von Frey and acetone tests. (C, F) Time course of single NTG‐induced migraine‐like allodynia is measured with the Von Frey test in male (C) and female (F) mice. (D, E, G, H) Time course of recurrent NTG‐induced migraine‐like allodynia is measured with the Von Frey (D, G) and acetone (E, H) tests in male (D, E) and female (G, H) mice. (I and J) Representative images and graphs of c‐Fos‐positive neurons in the PVN of male (I) and female (J) mice after five NTG or vehicle injections. Scale bars, 200 µm (left) and 100 µm (right). (K and L) Representative images (K) and graphs (L) showing the colocalization of CRF mRNA, OXT mRNA and c‐Fos mRNA. Scale bars, 100 µm. All values are presented as the mean ± SEM. (C–H) ^**^ *p* < 0.01 vs. Vehicle+ConAb; ^#^ *p* < 0.05, ^##^ *p* < 0.01 vs. NTG+ConAb, Bonferroni *post‐hoc* test after repeated‐measurement ANOVA (C: Groups, F_(2,21)_ = 20.428, *p* < 0.001; Time, F_(2,21)_ = 17.741, *p* < 0.001; interaction, F_(2,21)_ = 8.921, *p* < 0.001; D. Groups, F_(2,21)_ = 57.713, *p* < 0.001; Time, F_(2,21)_ = 9.126, *p* < 0.001; interaction, F_(2,21)_ = 5.500, *p* < 0.001; E. Groups, F_(2,21)_ = 61.318, *p* < 0.001; Time, F_(2,21)_ = 17.837, *p* < 0.001; interaction, F_(2,21)_ = 8.426, *p* < 0.001; F: Groups, F_(2,21)_ = 33.782, *p* < 0.001; Time, F_(2,21)_ = 13.494, *p* < 0.001; interaction, F_(2,21)_ = 9.412, *p* < 0.001; G: Groups, F_(2,21)_ = 43.785, *p* < 0.001; Time, F_(2,21)_ = 12.893, *p* < 0.001; interaction, F_(2,21)_ = 5.380, *p* < 0.001; H: Groups, F_(2,21)_ = 48.653, *p* < 0.001; Time, F_(2,21)_ = 10.261, *p* < 0.001; interaction, F_(2,21)_ = 6.668, *p* < 0.001). L. ^**^ *p* < 0.01 vs. Vehicle, **^##^** *p* < 0.01 vs. c‐Fos+OXT+ neurons/OXT+ neurons, Bonferroni post hoc test after two‐way ANOVA (NTG, F_(1,10)_ = 290.324, *p* < 0.001; c‐Fos+CRF+ neurons/CRF+ neurons, F_(1,10)_ = 741.487, *p* < 0.001; interaction, F_(1,10)_ = 658.792, *p* < 0.001). (I and J) ^*^ *p* < 0.05, ^**^ *p* < 0.01 vs. Vehicle, two‐tailed t‐test.

To establish a chronic migraine model in mice, we employed an intermittent intraperitoneal (i.p.) injection protocol of NTG (10 mg/kg, every 48 h for a total of five injections) [15], with behavioral assessments performed on each injection day and one day following the final injection (Figure 1A). Mice were habituated for 1 week prior to baseline testing. Von Frey and acetone tests were performed to assess the cephalic mechanical and cold allodynia, respectively (Figure 1B). We employed the von Frey test to measure cephalic cutaneous thresholds to mechanical stimuli at pre‐2 h (BL) and post‐1, ‐2, and ‐4 h of the first NTG injection on day 1 (Figure 1C,F). Then, migraine‐like allodynia was assessed on days 3, 5, 7, and 9 — 2 h before each NTG injection—and again on day 10. At these time points, both von Frey and acetone tests were performed to evaluate mechanical (Figure 1D,G) and cold allodynia (Figure 1E,H), respectively. Behavioral evaluation using von Frey filaments revealed a significant reduction in periorbital mechanical thresholds as early as 1 h after the first NTG injection in male and female mice, indicating acute mechanical allodynia (Figure 1C,F). This effect was transient, vanishing within 4 h post‐injection. Notably, both periorbital mechanical and cold allodynia were re‐identified during days 5–10 in male and female mice, indicating that repeated NTG exposure induces sustained migraine‐like periorbital allodynia with a time‐dependent trajectory (Figure 1D,E,G,H). Anti‐CGRP drugs have shown significant efficacy in the prophylactic treatment of episodic and chronic migraine [16, 17]. To further validate the translational value of the present model, we evaluated the prophylactic effect of a monoclonal antibody targeting CGRP, fremanezumab, in both acute and chronic migraine‐like allodynia paradigms. Given that fremanezumab exhibits a half‐life of 52.4 h in mice [18], we administered 30 mg/kg fremanezumab or an isotype‐matched control antibody (conAb) intraperitoneally on days 0, 4, and 8 to assess its preventive efficacy (Figure 1A). Behavioral assessments using von Frey filaments and acetone evaporation tests revealed that pretreatment with fremanezumab significantly attenuated NTG‐induced mechanical and cold allodynia in both male and female mice (Figure 1C–H), suggesting the NTG‐induced migraine model exhibits sufficient similarity to the symptoms observed in migraine patients.

To identify potential central mechanisms underlying these behavioral changes, we examined neuronal activation in the PVN via c‐Fos immunohistochemistry. Recurrent NTG‐treated male and female mice exhibited a striking increase in c‐Fos^+^ neurons within the PVN compared to vehicle‐injected controls (Figure 1I,J), with quantitative analysis revealing more than 4‐fold elevation in neuronal activity. These data implicate the PVN as a potential central hub in NTG‐induced migraine‐like conditions. Recent studies revealed the potential functions of PVN^OXT^ neurons in migraine regulation [14, 19]. However, our RNAscope fluorescent staining showed that repeated NTG injections induced c‐Fos mRNA expression at a significantly higher level in PVN^CRF^ neurons than in PVN^OXT^ neurons (Figure 1K,L). This discrepancy in neuronal activity between PVN^CRF^ neurons and PVN^OXT^ neurons was also demonstrated in transgenic mice with recurrent NTG treatment (Figure S1A–C). Therefore, the c‐Fos expression profile indicates that CRF‐expressing PVN neurons constitute the predominant activated population during the NTG‐induced migraine‐like conditions. Together, these findings validate the NTG protocol as a reliable model of chronic migraine that recapitulates both temporal dynamics and sensory phenotypes observed clinically. The observed activation of PVN^CRF^ neurons further highlights a potential central mechanism underlying migraine‐like allodynia.

### 2.2 NTG‐induced Migraine‐Like Allodynia Correlates With the Hyperactivity of the PVN^CRF^ Neurons

**FIGURE 2:**
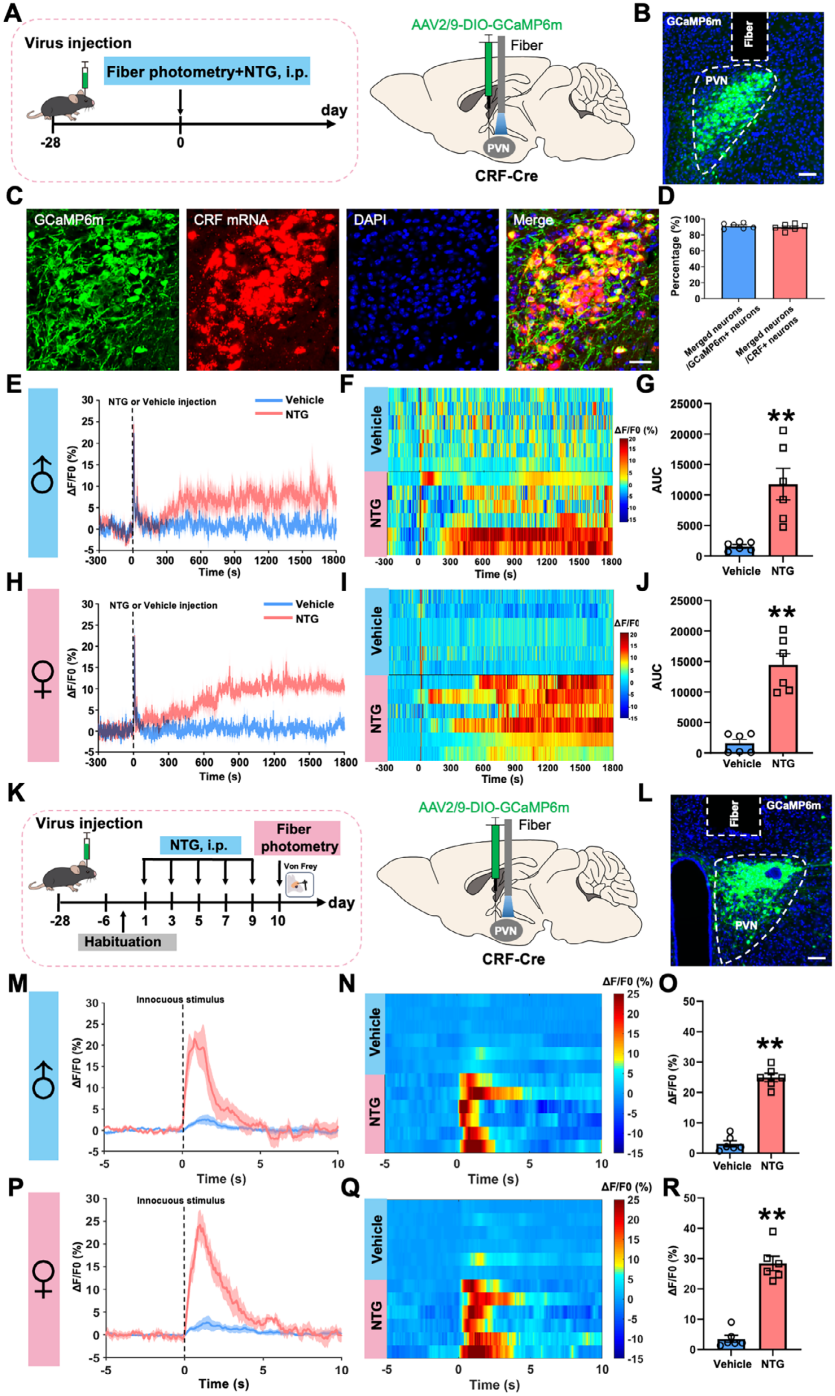
PVN^CRF^ neurons activation is related to NTG‐induced migraine‐like allodynia. (A) Experimental schedule for viral injection into the unilateral PVN with optical fiber implantation and NTG injection. (B) Frontal sections showing GCaMP6m‐labelled CRF neurons in the PVN. Scale bars, 100 µm. (C and D) Validating the specificity of the CRF‐Cre line and accuracy of viral injections. Scale bars, 100 µm. (E–J) Dynamics (E, H), heatmaps (F, I), and statistics (G, J) of PVN^CRF^ normalized Ca2^+^ activity in male (E–G) and female (H‐J) mice after an NTG injection. (K) Experimental schedule for viral injection, optical fiber implantation, repeated NTG injections, and behavioral tests. L. Frontal sections showing GCaMP6m‐labelled CRF neurons in the PVN. Scale bars, 100 µm. (M–R) Dynamics (M, P), heatmaps (N, Q), and statistics (O, R) of PVN^CRF^ normalized Ca2^+^ activity during innocuous stimuli in repeated NTG‐injected male (M‐O) and female (P‐R) mice. All values are presented as the mean ± SEM. ^**^ *p* < 0.01 vs. Vehicle, two tailed *t* test.

To dissect the functional contribution of CRF neurons in the PVN to migraine‐like allodynia, we first performed real‐time calcium imaging using in vivo fiber photometry (Figure 2A). CRF‐Cre mice were unilaterally injected with adeno‐associated virus AAV2/9‐DIO‐GCaMP6m into the PVN to enable specific monitoring of PVN^CRF^ neuronal activity. GCaMP6m expression was localized to the PVN region, confirming successful targeting (Figure 2B). Moreover, CRF‐targeted GCaMP6m fluorescence closely matched endogenous CRF mRNA expression, demonstrating that this Cre line can effectively target CRF neurons (Figure 2C,D). Upon systemic administration of NTG, we observed a robust and sustained elevation of calcium activity in PVN^CRF^ neurons relative to vehicle‐treated controls in male (Figure 2E–G) and female (Figure 2H–J) mice, indicating significant activation of this population under NTG‐induced migraine‐like conditions.

Given that cutaneous allodynia is recognized as a hallmark symptom of migraine conditions [20], we then examined the sensitivity of PVN^CRF^ neurons to transient allodynic stimuli. Four weeks after the AAV2/9‐DIO‐GCaMP6m injection into the unilateral PVN, we established the NTG‐induced chronic migraine model, followed by applying transient allodynic stimuli under fiber photometry (Figure 2K,L). In the NTG‐induced male and female murine migraine model, calcium signals of PVN^CRF^ neurons were rapidly increased in response to innocuous stimuli (0.07‐g von Frey filament) on day 10, suggesting PVN^CRF^ neurons are involved in migraine‐like allodynia (Figure 2M–R). Taken together, these results indicate that NTG‐induced migraine‐like allodynia correlates with the hyperactivity of the PVN^CRF^ neurons in both male and female mice.

### 2.3 Chemo‐inhibition of PVN^CRF^ Neurons Attenuates NTG‐induced Migraine‐Like Allodynia

**FIGURE 3:**
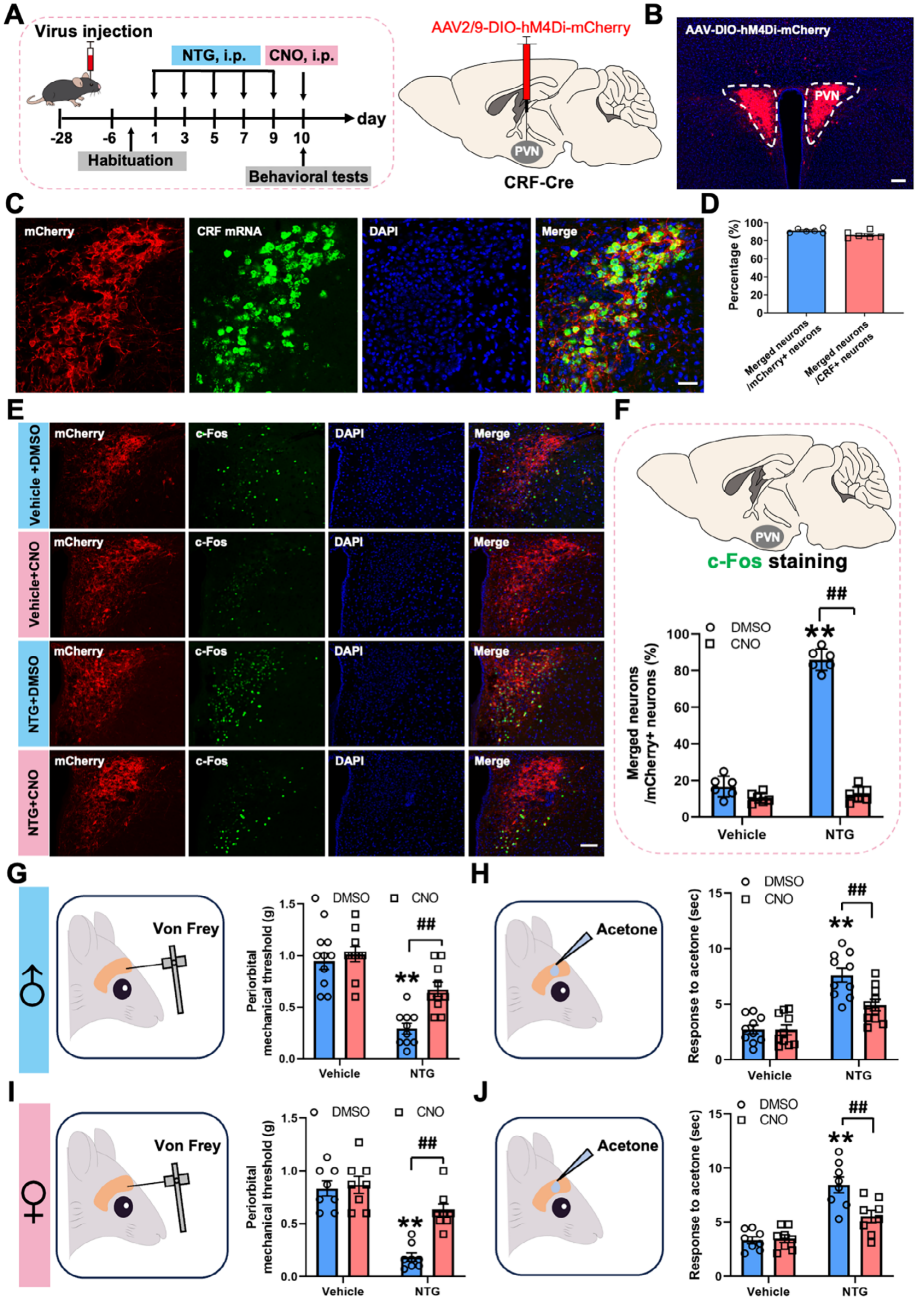
PVN^CRF^ neurons inhibition alleviates NTG‐induced migraine‐like allodynia. (A) Experimental schedule for viral injections into the PVN, NTG and CNO injections and behavioral tests. (B) Frontal sections showing mCherry‐expressing PVN^CRF^ neurons in the PVN. Scale bars, 200 µm. (C, D) Validating the specificity of the CRF‐Cre line and accuracy of viral injections. Scale bars, 100 µm. (E, F) Representative images (E) and graphs (F) of the overlap between PVN^CRF^ neurons and c‐Fos after CNO injection. Scale bars, 100 µm. (G–J) Von Frey (G, I) and acetone (H, J) tests showing effects of PVN^CRF^ neurons inhibition on periorbital hyperalgesia in CRF‐Cre male (G, H) and female (I, J) mice with repeated NTG injections. All values are presented as the mean ± SEM. ^**^ *p* < 0.01 vs. Vehicle+DMSO, ^##^ *p* < 0.01 vs. NTG+DMSO, Bonferroni post hoc test after two‐way ANOVA (F: NTG, F_(1,10)_ = 236.773, *p* < 0.001; CNO, F_(1,10)_ = 686.083, *p* < 0.001; interaction, F_(1,10)_ = 490.143, *p* < 0.001; G: NTG, F_(1,18)_ = 36.260, *p* < 0.001; CNO, F_(1,18)_ = 18.033, *p* < 0.001; interaction, F_(1,18)_ = 8.875, *p* = 0.008; H: NTG, F_(1,18)_ = 36.078, *p* < 0.001; CNO, F_(1,18)_ = 12.829, *p* = 0.002; interaction, F_(1,18)_ = 12.273, *p* = 0.003; I: NTG, F_(1,14)_ = 44.574, *p* < 0.001; CNO, F_(1,14)_ = 14.036, *p* = 0.002; interaction, F_(1,14)_ = 10.204, *p* = 0.006; J: NTG, F_(1,14)_ = 50.458, *p* < 0.001; CNO, F_(1,14)_ = 6.598, *p* = 0.022; interaction, F_(1,14)_ = 8.102, *p* = 0.013).

To test the necessity of PVN^CRF^ neuronal activity in NTG‐evoked responses, we applied a chemogenetic strategy using AAV2/9‐DIO‐hM4Di‐mCherry (AAV2/9‐DIO‐mCherry was used as a control) in CRF‐Cre mice to selectively inhibit the activities of PVN^CRF^ neurons (Figure 3A and Figure S2A). Viral expression was confined to the PVN, and mCherry fluorescence confirmed selective targeting of PVN^CRF^ neurons (Figure 3B; Figure S2B). RNAscope results showed that mCherry fluorescence closely matched endogenous CRF mRNA expression, demonstrating that this Cre line can effectively target CRF neurons (Figure 3C,D; Figure S2C,D). Immunohistochemical analysis of c‐Fos exhibited that the number of c‐Fos^+^/mCherry^+^ neurons was significantly reduced in NTG‐treated mice receiving clozapine‐N‐oxide (CNO, 2 mg/kg), compared to NTG‐treated mice treated with the same volume of 1%DMSO control (Figure 3E,F), indicating that CNO injection could potently inhibit the NTG‐induced activation of PVN^CRF^ neurons. To determine whether PVN^CRF^ neurons contribute to NTG‐induced migraine‐like allodynia, we conducted behavioral assays measuring both mechanical and cold allodynia 30 min after CNO or 1%DMSO administration on day 10. NTG‐treated male and female mice exhibited significantly reduced periorbital mechanical thresholds (Figure 3G,I) and prolonged response durations to acetone (Figure 3H,J), indicating the occurrence of both mechanical and cold allodynia. These changes closely paralleled those observed in NTG‐induced chronic migraine models [15]. Importantly, chemogenetic inhibition of PVN^CRF^ neurons via CNO significantly reversed both phenotypes in male and female mice (Figure 3G–J; Figure S2E,F), suggesting that the activation of PVN^CRF^ neurons is required for the expression of migraine‐related allodynia in both male and female mice.

### 2.4 Chemo‐Activation of PVNCRF Neurons Recapitulates Migraine‐Like Allodynia

**FIGURE 4:**
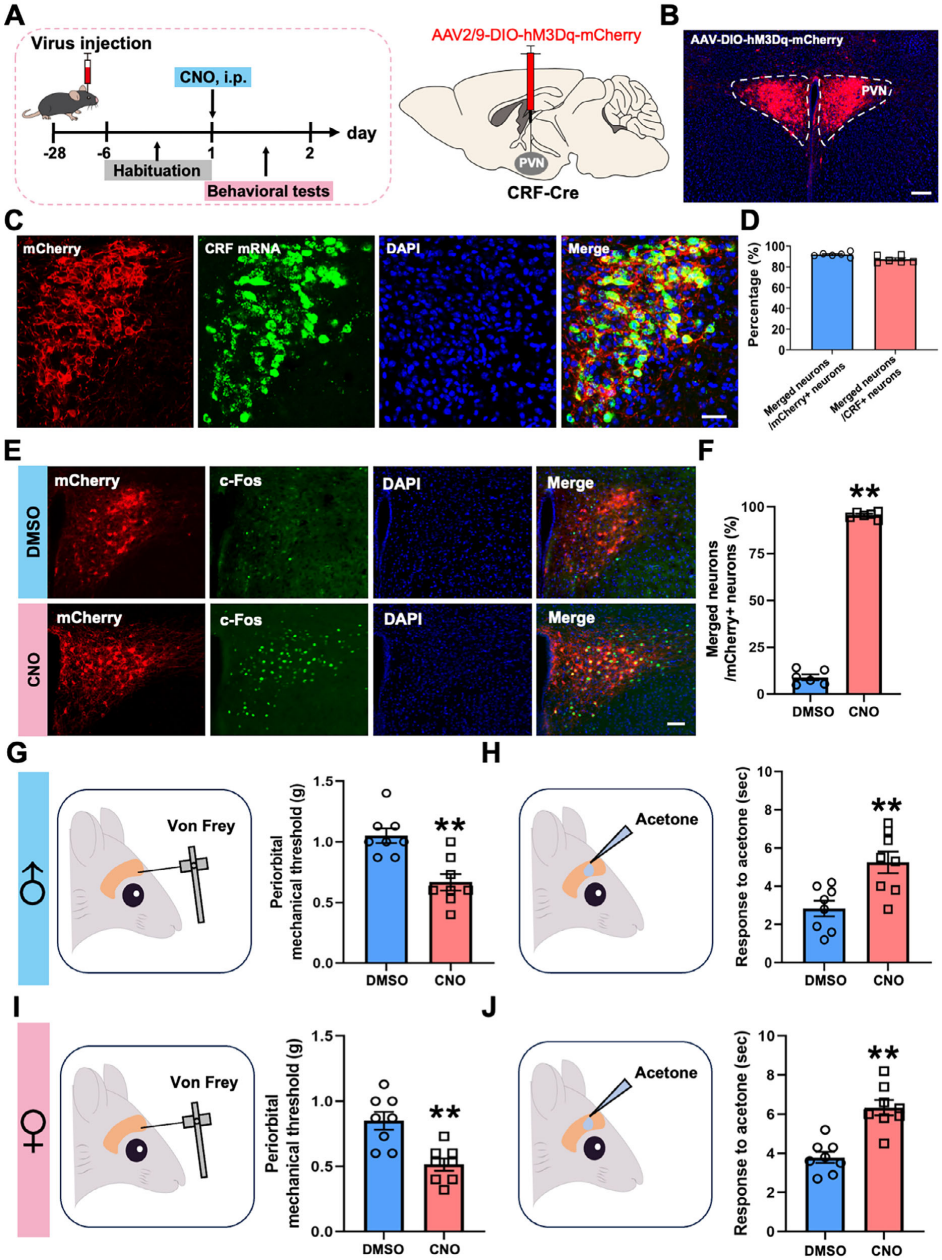
PVN^CRF^ neurons activation induced migraine‐like allodynia. (A) Experimental schedule for viral injections into the PVN, CNO injections, and behavioral tests. (B) Frontal sections showing mCherry‐expressing PVN^CRF^ neurons in the PVN. Scale bars, 200 µm. (C, D) Validating the specificity of the CRF‐Cre line and the accuracy of viral injections. Scale bars, 100 µm. (E, F) Representative images (E) and graphs (F) of the overlap between PVN^CRF^ neurons and c‐Fos after CNO injection. Scale bars, 100 µm. (G–J) Von Frey (G, I) and acetone (H, J) tests showing effects of PVN^CRF^ neurons activation on periorbital mechanical and thermal sensation in CRF‐Cre male (G, H) and female (I, J) mice. All values are presented as the mean ± SEM. ^**^ *p *< 0.01 vs. DMSO, two‐tailed t‐test.

To assess the sufficiency of PVN^CRF^ neurons in mediating migraine‐like allodynia, we performed chemogenetic activation using a Cre‐dependent viral strategy. AAV2/9‐DIO‐hM3Dq‐mCherry (AAV2/9‐DIO‐mCherry was used as a control) was stereotaxically injected into the PVN of CRF‐Cre mice to specifically activate PVN^CRF^ neurons. After a 28‐day postsurgical recovery period to ensure stable transgene expression, behavioral testing was conducted 30 min after systemic administration of CNO (2 mg/kg) via i.p. (Figure 4A and Figure S2A). The mCherry expression pattern and the RNAscope staining validated the accuracy and specificity of viral transfection (Figure 4B–D; Figure S2B–D). CNO administration robustly induced c‐Fos expression in hM3Dq‐mCherry^+^ PVN neurons, indicating effective neuronal activation (Figure 4E,F). Behaviorally, Von Frey filament testing revealed a significant decrease in periorbital mechanical threshold in CNO‐treated male and female mice compared to 1%DMSO controls (Figure 4G,I), indicating the presence of mechanical allodynia. Concurrently, CNO‐treated mice exhibited significantly prolonged nocifensive responses in the acetone‐induced cold allodynia assay (Figure 4H,J), reflecting heightened cold sensitivity. These changes closely paralleled those observed in NTG‐induced chronic migraine models. Importantly, 1%DMSO‐injected mice exhibited neither neuronal activation nor altered basal sensation (Figure 4E–J; Figure S2E,F), confirming that the observed cephalic cutaneous allodynia was specifically driven by chemogenetic stimulation of PVN^CRF^ neurons. Together, these results demonstrate that selective activation of PVN^CRF^ neurons is sufficient to reproduce core features of migraine‐like allodynia in both male and female mice, underscoring their functional role in migraine sensitization.

### 2.5 PVN^CRF^ Neurons Modulate Migraine‐Like Allodynia Through Their Output to SP5C

The above‐described results established that PVN^CRF^ neurons were both necessary and sufficient to drive migraine‐like allodynia. However, the specific downstream targets responsible for conveying this effect remain unclear. Previous studies have demonstrated that the SP5C serves as a critical hub in the regulation of trigeminal nociceptive transmission, receiving direct neural projections from PVN neurons [21]. Therefore, we aimed to investigate whether PVN^CRF^ neurons could modulate migraine‐like allodynia through innervating the SP5C. Using multiplex in situ hybridization combined with immunofluorescence to assess co‐expression of neuropeptides in retrogradely labeled cells, we found that NTG injection could significantly activate the neurons of SP5C (Figure S3A,B), and 78.3 ± 4.2% of EGFP^+^ PVN neurons (retrogradely labeled from the SP5C) co‐expressed CRF mRNA, while approximately 21.6 ± 3.1% expressed OXT mRNA (Figure S3C–F), indicating a predominant CRFergic phenotype among SP5C‐projecting PVN neurons.

**FIGURE 5:**
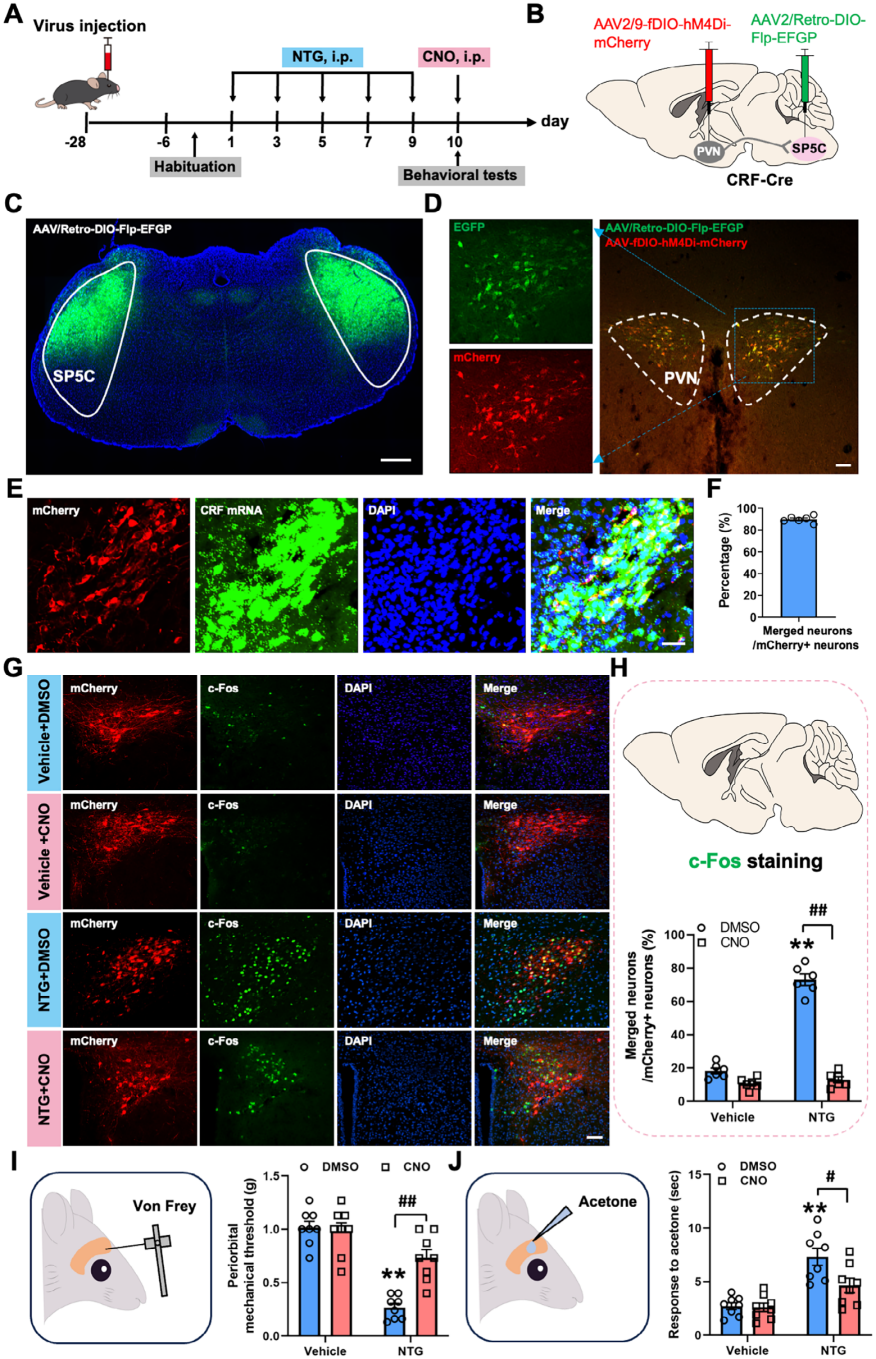
PVN^CRF^ neurons output to SP5C to regulate NTG‐induced migraine‐like allodynia. (A, B) Experimental schedule for viral injections into the PVN, NTG, and CNO injections and behavioral tests. (C) Frontal sections showing EGFP‐expressing neurons in the SP5C. Scale bars, 200 µm. (D) Frontal sections showing EGFP‐ and mCherry‐expressing neurons in the PVN. Scale bars, 100 µm. (E, F) Validating the specificity of the CRF‐Cre line and the accuracy of viral injections. Scale bars, 100 µm. (G, H) Representative images (G) and graphs (H) of the overlap between PVN^CRF^ neurons and c‐Fos after CNO injection. Scale bars, 100 µm. (I, J) Von Frey (I) and acetone (J) tests showing effects of SP5C‐projecting PVN^CRF^ neurons inhibition on periorbital hyperalgesia in CRF‐Cre mice with repeated NTG injections. All values are presented as the mean ± SEM. ^**^ *p* < 0.01 vs. Vehicle+DMSO, ^#^ *p* < 0.05, ^##^ *p* < 0.01 vs. NTG+DMSO, Bonferroni *post‐hoc* test after two‐way ANOVA (H: NTG, F_(1,10)_ = 429.794, *p* < 0.001; CNO, F_(1,10)_ = 137.588, *p *< 0.001; interaction, F_(1,10)_ = 83.277, *p *<  0.001; I: NTG, F_(1,14)_ = 45.382, *p *< 0.001; CNO, F_(1,14)_ = 15.915, *p *= 0.001; interaction, F_(1,14)_ = 21.271, *p *< 0.001; J: NTG, F_(1,14)_ = 46.022, *p *< 0.001; CNO, F_(1,14)_ = 4.500, *p *= 0.052; interaction, F_(1,14)_ = 3.803, *p *= 0.071).

To verify whether the SP5C‐projecting PVN^CRF^ neurons are involved in migraine‐like allodynia, CRF‐Cre mice were injected with AAV2/Retro‐hSyn‐DIO‐Flp‐EGFP into bilateral SP5C, followed by AAV2/9‐EF1a‐fDIO‐hM4Di‐mCherry injection into bilateral PVN (Figure 5A,B). This strategy allowed for specific expression of hM4Di‐mCherry in PVN^CRF^ neurons with direct monosynaptic projections to the SP5C. 28 days postsurgery, animals were subjected to the established NTG‐induced chronic migraine protocol. On day 10, CNO was administered via i.p. to selectively inhibit SP5C‐projecting PVN^CRF^ neurons. Dual fluorescence labeling revealed EGFP^+^/mCherry^+^ neurons (yellow color) in the PVN, representing the targeted SP5C‐projecting PVN^CRF^ population (Figure 5C,D). In addition, RNAscope staining showed almost mCherry‐labeled neurons were CRF immunoreactive, suggesting the specificity of chemogenetic modulation (Figure 5E,F). Importantly, CNO administration markedly reduced c‐Fos expression within these projection‐defined PVN^CRF^ neurons (Figure 5G,H), demonstrating effective and pathway‐specific chemogenetic inhibition. Behavioral assays further established the functional role of this projection in mediating migraine‐like allodynia. NTG‐treated animals exhibited significant periorbital mechanical allodynia and cold allodynia (Figure 5I,J). However, chemo‐inhibition of SP5C‐projecting PVN^CRF^ neurons significantly reversed both mechanical and cold hypersensitivity (Figure 5I,J). These findings identify the PVN^CRF^‐SP5C circuit as a critical efferent pathway required for the expression of NTG‐induced migraine‐like allodynia.

### 2.6 SP5C glutamatergic Neurons Input From PVN^CRF^ Mediates Migraine‐Like Allodynia

**FIGURE 6:**
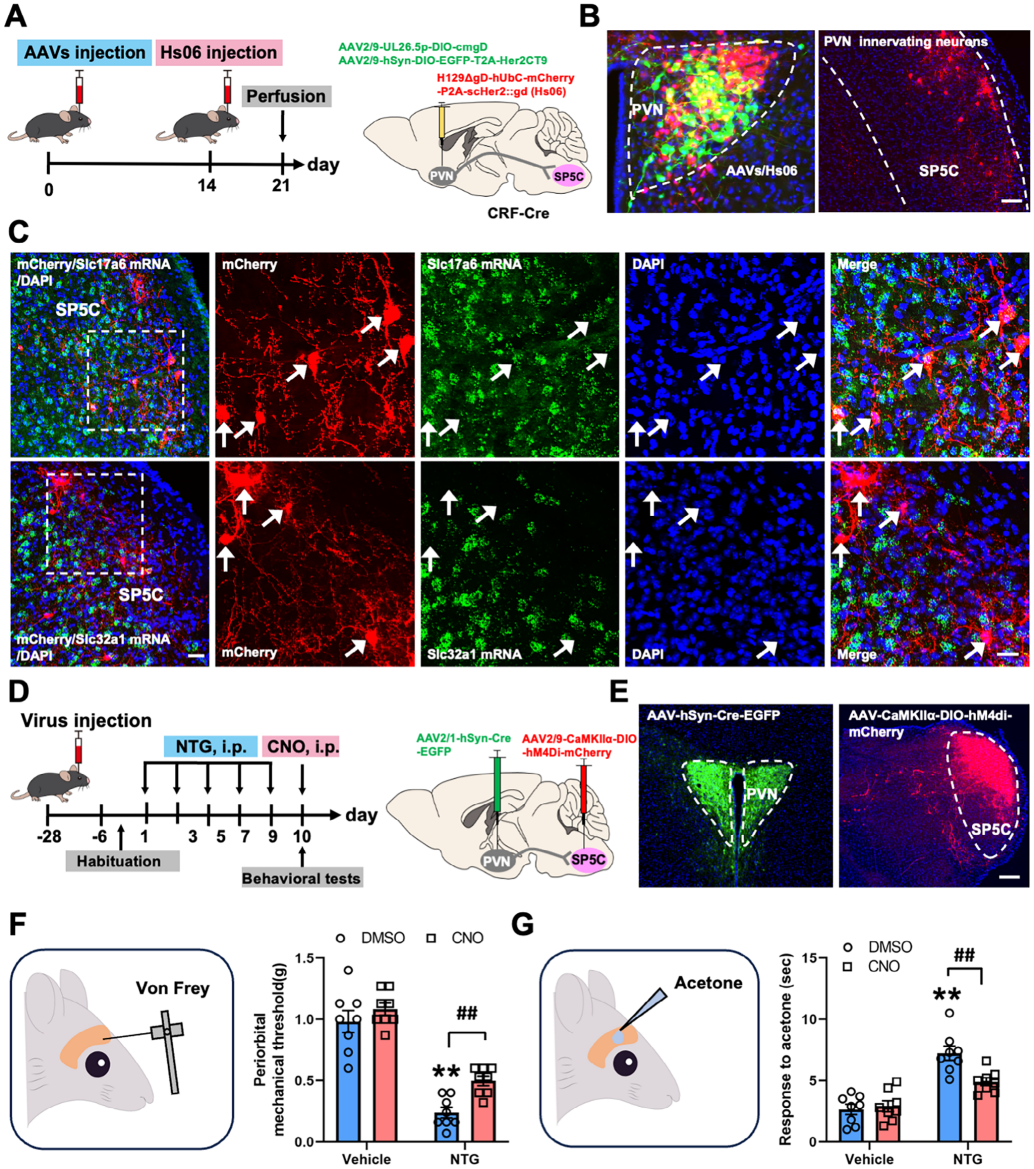
SP5C^Glu^ neurons innervated by PVN^CRF^ participate in NTG‐induced migraine‐like allodynia. (A) Viral injection strategy for labeling PVN^CRF^ neurons and their downstream targets. (B) Frontal sections showing EGFP‐ and mCherry‐expressing neurons in the PVN and mCherry‐expressing neurons in the SP5C. Scale bars, 200 µm. (C) RNAscope staining showing that mCherry neurons are colocalized with glutamate but not GABA in the SP5C. White arrows indicate mCherry‐expressing neurons. Scale bar, 100 µm (left) and 20 µm (right). (D) Experimental schedule for viral injections into the PVN and SP5C, NTG and CNO injections, and behavioral tests. (E) Frontal sections showing EGFP‐expressing neurons in the PVN and mCherry‐expressing neurons in the SP5C. Scale bars, 200 µm. (F and G) Von Frey (F) and acetone (G) tests showing effects of PVN‐innervated SP5C^Glu^ neurons inhibition on periorbital hyperalgesia in mice with repeated NTG injections. All values are presented as the mean ± SEM. ^**^ *p* < 0.01 vs. Vehicle+DMSO, ^##^ *p* < 0.01 vs. NTG+DMSO, Bonferroni post hoc test after two‐way ANOVA (F: NTG, F_(1,14)_ = 128.919, *p *< 0.001; CNO, F_(1,14)_ = 9.372, *p *= 0.008; interaction, F_(1,14)_ = 1.794, *p *= 0.202; G: NTG, F_(1,14)_ = 32.454, *p *< 0.001; CNO, F_(1,14)_ = 16.625, *p *= 0.001; interaction, F_(1,14)_ = 25.663, *p *< 0.001).

To delineate the anatomical architecture and functional contribution of PVN^CRF^ projections to the SP5C, we established a Cre‐dependent, monosynaptic anterograde viral tracing system in CRF‐Cre mice [22]. AAV2/9‐UL26.5P‐DIO‐cmgD and AAV2/9‐hSyn‐DIO‐EGFP‐T2A‐HER2CT9 were co‐injected into the PVN to selectively express a synthetic glycoprotein D (cmgD) and the truncated human epidermal growth factor receptor variant HER2CT9, respectively, in CRF‐expressing neurons. Both constructs were designed with a double‐floxed inverse orientation (DIO) configuration to confer Cre specificity, and expression was driven by the human synapsin (hSyn) promoter, ensuring neuron‐selective targeting. Fourteen days post‐injection, a modified gD‐deleted herpes simplex virus (HSV) strain, H129ΔgD‐hUbc‐mCherry‐P2A‐scHer2::gD (HS06), was introduced into the same site. This engineered virus utilizes cmgD and/or HER2CT9 for cell entry and anterograde transsynaptic spread, enabling precise labeling of postsynaptic targets downstream of PVN^CRF^ neurons [23]. EGFP expression confirmed transduction of starter cells in the PVN, while a robust mCherry signal was observed in the SP5C, indicating a direct, monosynaptic projection from PVN^CRF^ neurons to the SP5C (Figure 6A,B). To determine the neurotransmitter identity of SP5C recipient neurons, we performed immunohistochemical co‐staining for the excitatory marker Slc17a6 (Vglut2) and inhibitory marker Slc32a1 (Vgat). Quantification revealed that the majority of mCherry^+^ neurons in SP5C co‐expressed Slc17a6, with negligible overlap with Slc32a1(Figure 6C), indicating that SP5C neurons innervated by PVN^CRF^ are glutamatergic excitatory neurons (SP5C^Glu^). To evaluate the functional relevance of this projection in the context of migraine‐like allodynia, we implemented a dual‐site chemogenetic inhibition strategy targeting the PVN‐SP5C^Glu^ axis. AAV2/1‐hSyn‐Cre‐EGFP was delivered to the PVN to enable anterograde Cre transport, while AAV2/9‐CaMKIIα‐DIO‐hM4Di‐mCherry was injected into SP5C to induce Cre‐dependent expression of the inhibitory DREADD receptor hM4Di selectively in SP5C^Glu^ neurons innervated by PVN (Figure 6D). Confined GFP‐labeled PVN neurons and mCherry‐labeled SP5C^Glu^ neurons were observed in mice with virus injection, indicating that the targeted injection sites were accurate (Figure 6E). Following repeated administration of NTG (10 mg/kg, i.p.), mice developed persistent cephalic allodynia, which was significantly attenuated by chemogenetic inhibition of PVN‐innervating SP5C^Glu^ using CNO (2 mg/kg, i.p.) (Figure 6F,G). These results together indicate that SP5C^Glu^ neurons innervated by PVN^CRF^ neurons participate in migraine‐like allodynia.

Extensive studies have established that the anterior cingulate cortex (ACC) and insular cortex (IC) are critically involved in pain sensitization [24, 25, 26]. Given our findings demonstrating that manipulation of the PVN^CRF^‐SP5C^Glu^ circuit significantly modulates migraine‐like cutaneous allodynia, we sought to examine whether the activities of ACC/IC are also regulated by PVN‐SP5C pathways in this NTG‐induced migraine‐like condition. To this end, CRF‐Cre mice received bilateral injections of AAV2/Retro‐hSyn‐DIO‐Flp‐EGFP into the SP5C, followed by bilateral AAV2/9‐EF1a‐fDIO‐hM4Di‐mCherry injections into the PVN. This dual‐viral approach enabled selective chemogenetic inhibition of SP5C‐projected PVN^CRF^ neurons (Figure S4A,B). Our results demonstrate that repeated NTG injections significantly enhanced neuronal activities in ACC/IC, while chemogenetic inhibition of the PVN‐SP5C pathway blocked these ACC/IC activations (Figure S4C,D), indicating that ACC/IC activation in the NTG‐induced migraine‐like state is modulated by PVN‐SP5C pathways. However, using dual retrograde AAV tracers injected separately into ACC/IC and SP5C (Figure S4E,H), we identified that SP5C‐projecting PVN neurons were spatially distinct from ACC/IC‐projecting PVN neurons (Figure S4F,G,I,J), which suggests that PVN SP5C‐projecting neurons do not directly regulate ACC/IC neurons to modulate migraine‐like behaviors, but rather indirectly influence the ascending signaling from SP5C to ACC/IC for pain regulation.

### 2.7 CRF Neuropeptide in the SP5C Increases After NTG Injection and Facilitates Trigeminal Nociceptive Transmission

**FIGURE 7:**
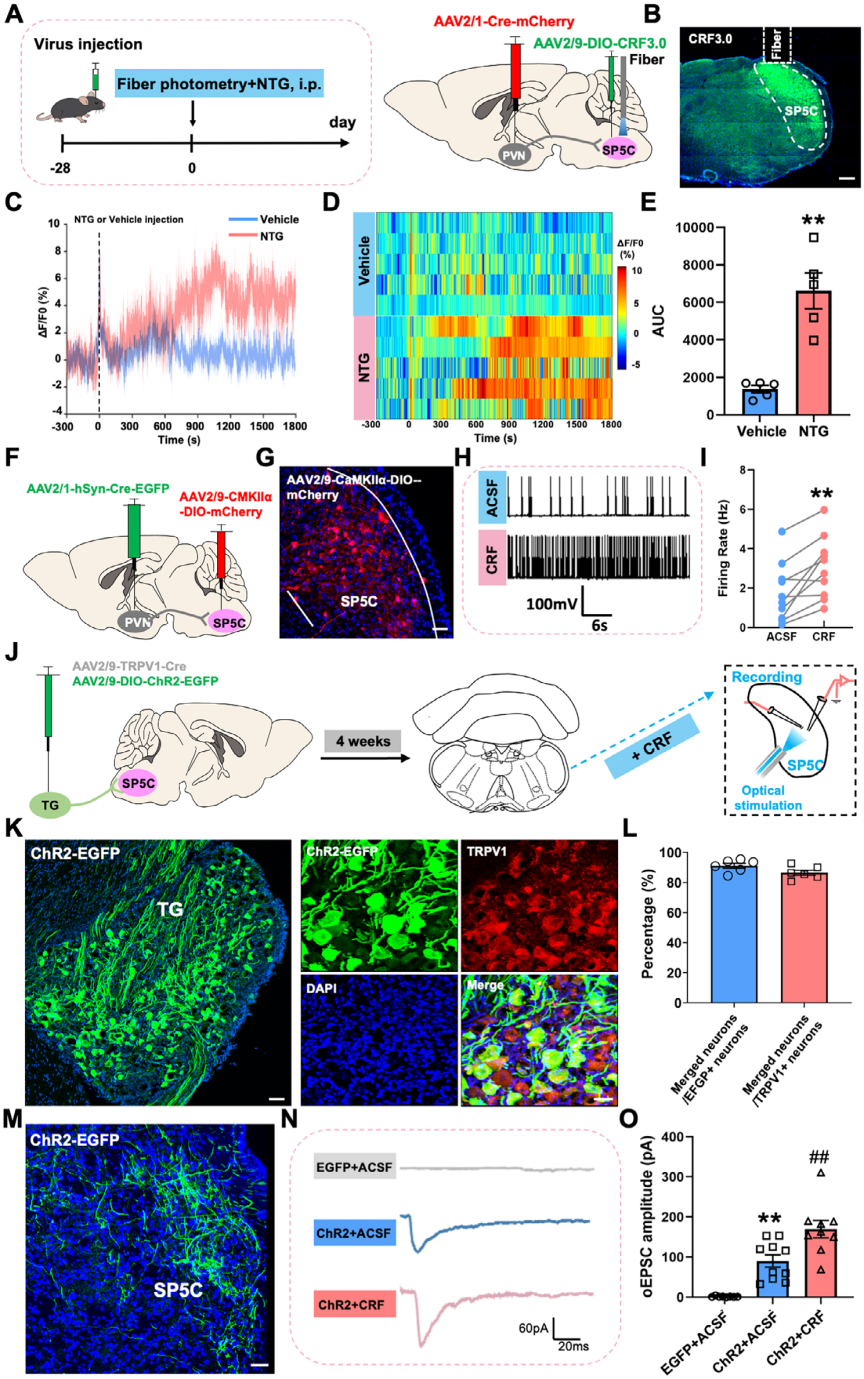
NTG injection‐induced CRF release facilitates neuronal excitability in the SP5C. (A) Experimental schedule for viral injections into unilateral PVN and SP5C with optical fiber implantation and NTG injections. (B) Frontal sections showing CRF3.0‐labelled neurons in the SP5C. Scale bars, 200 µm. (C–E) Dynamic (C), heatmap (D) and statistic (E) of SP5C normalized CRF activity after an NTG injection. (F) Experimental scedule for a PVN‐SP5C circuit viral injections and electrophysiological recording in the SP5C on brainstem slices. (G) Frontal sections showing mCherry‐expressing neurons in the SP5C. Scale bars, 100 µm. (H, I) Patch clamp showing that exogenous CRF significantly increases the firing rate of SP5C neurons. (J) Experimental schedule for optogenetic viral injections and electrophysiological recording in the SP5C on brainstem slices. (K, L) Validating the specificity of TRPV1‐Cre and the accuracy of viral injections. (M) Frontal sections showing ChR2‐expressing projections in the SP5C. Scale bars, 100 µm. (N, O) Patch clamp showing that exogenous CRF significantly increases the amplitudes of TG^TRPV1^ projections‐elicit oEPSC in the SP5C neurons. TG: trigeminal ganglion. All values are presented as the mean ± SEM. (E, I) ^**^ *p* < 0.01 vs. Vehicle or ACSF, two‐tailed *t* test. (O) ^**^ *p* < 0.01 vs. EGFP+ACSF, ^##^ *p* < 0.01 vs. ChR2+ACSF, Bonferroni post hoc test after one‐way ANOVA, F _(2,24)_ = 26.131, *p* < 0.001.

Given that CRF neurons transmit information by releasing CRF neuropeptide to downstream brain regions upon activation [27], and our findings confirm involvement of PVN^CRF^‐SP5C^Glu^ circuits in NTG‐induced migraine‐like conditions, we next investigate whether PVN^CRF^ neurons activate SP5C neurons through CRF neuropeptide signaling. To this end, we employed the newly developed CRF neuropeptide sensor, which consists of a fluorescent protein inserted into the CRF receptor [28]. When CRF binds to this receptor, it triggers a conformational change that alters the fluorescence intensity of the sensor, allowing real‐time monitoring of neuropeptide signaling dynamics in the SP5C region following NTG administration. AAV2/1‐hSyn‐Cre‐mCherry was unilaterally injected into PVN, and AAV2/9‐CaMKII‐DIO‐CRF3.0 was ipsilaterally injected into the SP5C to label the SP5C^Glu^ neurons that receive PVN projections and record CRF release dynamics following systemic injection of NTG or vehicle (Figure 7A). The viral expression and optic fiber placement were confirmed histologically (Figure 7B). Compared with vehicle‐treated controls, NTG administration led to a robust increase of CRF neuropeptide in SP5C (Figure 7C–E), suggesting that the release of CRF neuropeptide in the PVN‐ SP5C^Glu^ circuit increases after NTG injection.

To further verify whether CRF neuropeptide directly activates SP5C^Glu^ neurons, we electrophysiologically investigated its effects on neuronal excitability within SP5C. We specifically targeted PVN‐innervating SP5C^Glu^ neurons by delivering AAV2/1‐hSyn‐Cre‐EGFP into the PVN and AAV2/9‐CaMKIIα‐DIO‐mCherry into the SP5C via bilateral intracranial injections (Figure 7F). Confined mCherry‐labeled SP5C neurons were observed in mice with virus injections (Figure 7G), indicating that the virus was accurately targeted. Four weeks after the virus injection, we performed whole‐cell current‐clamp recordings in acute brainstem slices containing the SP5C. Bath application of CRF (200 nmol/L) resulted in a marked increase in spontaneous action potential firing compared to baseline recordings. Representative traces illustrate the heightened firing activity following CRF exposure (Figure 7H). Across recorded cells, CRF significantly elevated the mean firing rate relative to ACSF control conditions (Figure 7I), indicating a direct excitatory effect of CRF signaling on SP5C^Glu^ neurons. Since potentiation of excitatory synaptic transmissions in the SP5C underlies the sensitization of trigeminal nociceptive transmission [29], we subsequently examined whether CRF could enhance these synaptic transmissions. To achieve this, we employed an optogenetic approach to selectively express ChR2 in TRPV1‐positive trigeminal ganglion (TG) neurons that sense noxious stimuli and transmit pain signals [30]. Accordingly, we injected a mixture containing AAV2/9‐TRPV1‐Cre and AAV2/9‐DIO‐ChR2‐EGFP into the TG. After a 4‐week recovery period, we performed optogenetic manipulation and electrophysiological recording (Figure 7J). The majority of GFP‐labeled TG neurons expressed TRPV1, indicating the accuracy and specificity of viral targeting (Figure 7K–M). Electrophysiologically, photo‐stimulation (473 nm blue laser, 20 Hz, 10 ms pulses) of ChR2 in TG TRPV1‐expressing projections within the SP5C induced a significant optically‐evoked EPSC (oEPSC) in postsynaptic neurons (Figure 7N,O). As expected, the amplitudes of oEPSCs were significantly increased by CRF perfusion (Figure 7N,O), indicating the facilitative effect of CRF on the trigeminal nociceptive transmission at the SP5C level.

Taken together, these results demonstrate that under NTG‐induced migraine‐like conditions, PVN^CRF^ neurons drive the sensitization of trigeminal nociceptive transmission in the SP5C through enhanced CRF neuropeptide release.

### 2.8 The CRF Neuropeptide Activates CRFR2 Rather Than CRFR1 in SP5C to Participate in the Regulation of Migraine‐Like Allodynia

**FIGURE 8:**
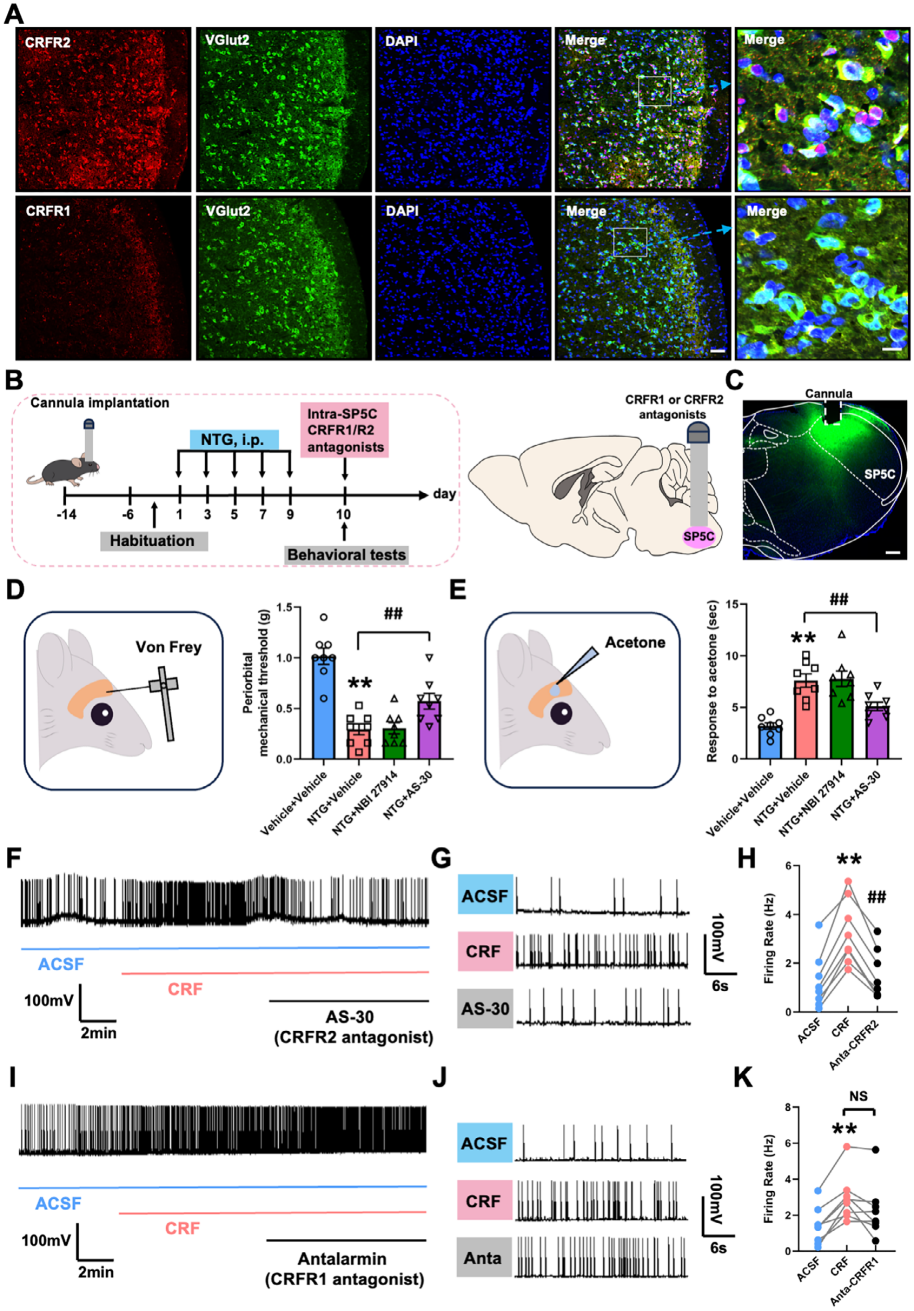
The CRF neuropeptide activates CRFR2 rather than CRFR1 in SP5C to regulate migraine‐like allodynia. (A) The immunostaining showing the expression and distribution patterns of CRFR1 and CRFR2 in SP5C. Scale bars, 100 µm (left) and 20 µm (right). (B) Experimental schedule for cannula implantation above bilateral SP5C, CRFR antagonist injections, and behavioral tests. C. Representative images showing cannula tracks above SP5C. The green fluorescent CTB‐488 dye enables visualization of injection sites. Scale bars, 200 µm. (D, E) Von Frey (D) and acetone (E) tests showing effects of antagonizing the SP5C CRF receptors on periorbital hyperalgesia in mice with repeated NTG injections. (F‐H) Patch clamp showing that the CRFR2 antagonist AS‐30 blocks the exogenous CRF‐evoked SP5C neuronal excitation. (I–K) Patch clamp showing that the CRFR1 antagonist antalarmin has no effect on the exogenous CRF‐evoked SP5C neuronal excitation. All values are presented as the mean ± SEM. ^**^ *p *< 0.01 vs. Vehicle+Vehicle or ACSF; ^##^ *p* < 0.01 vs. NTG+vehicle or CRF, Bonferroni post hoc test after one‐way ANOVA (D: F_(3,28)_ = 24.597, *p *< 0.001; E: F_(3,28)_ = 15.634, *p* < 0.001; H: F_(2,21)_ = 7.224, *p *= 0.0041; K: F_(2,21)_ = 2.931, *p *= 0.0754).

The CRF receptors mainly include two types: CRF receptor type 1 (CRFR1) and type 2 (CRFR2). Among them, CRFR1 is mainly located in the central nervous system, such as the pituitary gland and the cortex [31, 32], while CRFR2 is mainly located in peripheral tissues such as the cardiovascular system and the gastrointestinal tract [33, 34]. There are significant differences in their distribution and functional regulation [35]. Having confirmed that the CRF neuropeptide released by PVN^CRF^ neurons participates in the regulation of migraine‐like allodynia by activating SP5C^Glu^ neurons, we next investigated which type of receptor is involved in this process. To address this question, we first performed double‐label immunofluorescence for CRFR1, CRFR2, and the excitatory neuronal marker Vglut2. CRFR2 immunoreactivity was clearly detected in the SP5C and showed substantial colocalization with Vglut2‐positive excitatory neurons (Figure 8A). In contrast, CRFR1 expression in this region was minimal to undetectable (Figure 8A), indicating that CRFR2 is the predominant CRF receptor subtype in excitatory SP5C neurons. To assess the functional role of CRFR2 signaling in vivo, we employed an NTG‐induced chronic migraine model coupled with bilateral intra‐SP5C microinjections of selective CRFR antagonists via pre‐implanted cannulas (Figure 8B,C). Behavioral responses to mechanical and cold stimuli were evaluated on day 10. NTG‐treated mice exhibited significant reductions in periorbital mechanical thresholds and heightened cold sensitivity compared to vehicle‐injected controls (Figure 8D,E). Notably, intra‐SP5C administration of the CRFR2 antagonist Antisauvagine‐30 (AS‐30) significantly alleviated both mechanical and cold allodynia, whereas the CRFR1 antagonist Antalarmin had no significant effect (Figure 8D,E). These findings underscore the functional relevance of CRFR2 in mediating NTG‐induced migraine‐like allodynia. To further validate the role of CRFR2 in neuronal excitability, we conducted ex vivo whole‐cell current‐clamp recordings from neurons in acute brainstem slices containing the SP5C. Application of CRF markedly increased spontaneous firing activity in SP5C neurons compared to baseline (Figure 8F–H). This CRF‐induced increase in excitability was effectively abolished by pre‐treatment with AS‐30 (Figure 8F–H), confirming the involvement of CRFR2. By contrast, application of the CRFR1 antagonist Antalarmin did not significantly alter the excitatory response to CRF (Figure 8I–K), consistent with the absence of CRFR1 expression in this region. Together, these results identify CRFR2‐expressing excitatory neurons in the SP5C^Glu^ as a key substrate for CRF‐mediated facilitation of nociceptive signaling and further implicate CRFR2 as a critical mediator of NTG‐induced migraine‐like allodynia.

### 2.9 fMRI Revealed Significant Alterations in the PVN and SP5C Regions of Migraine Patients

**FIGURE 9:**
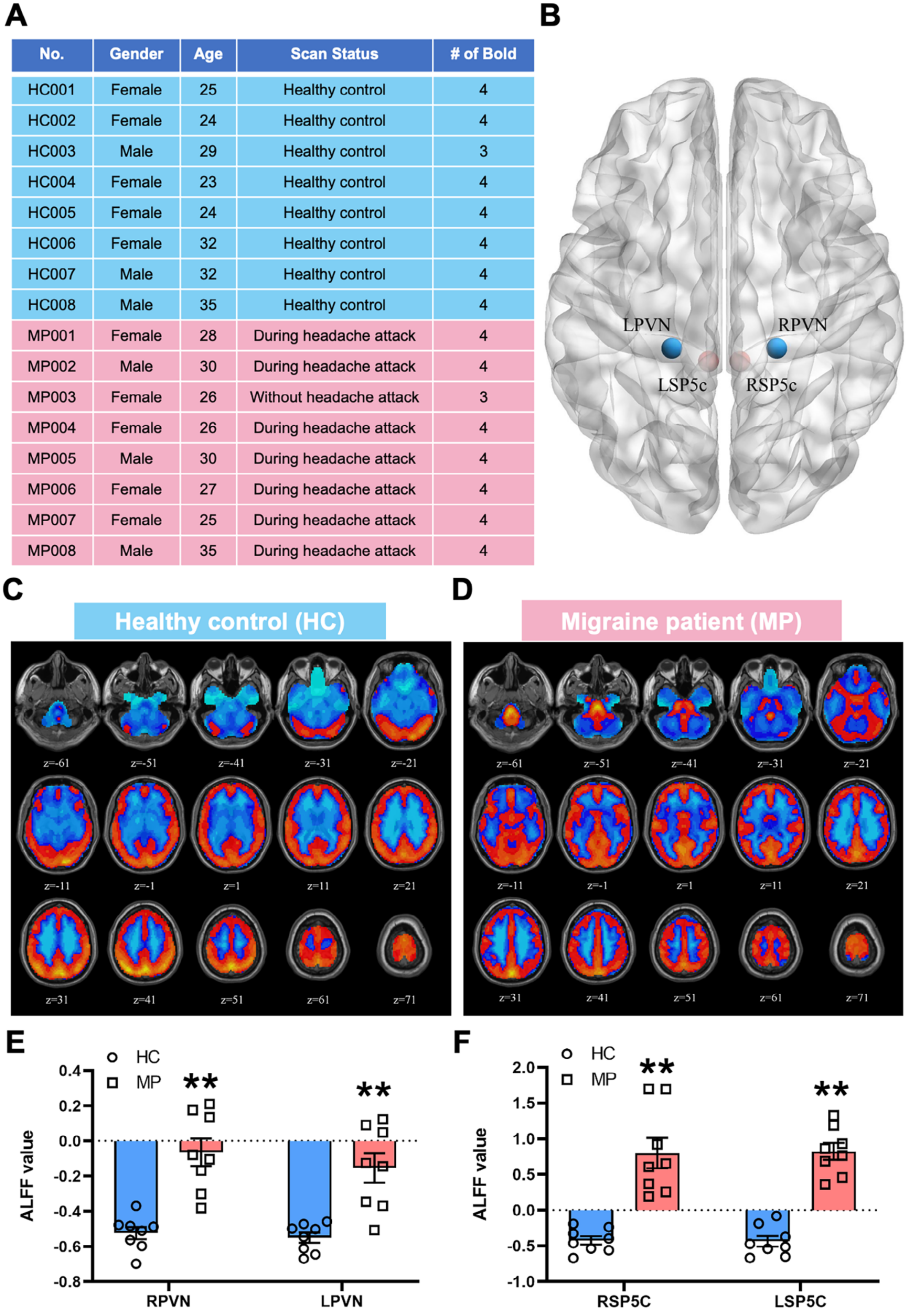
fMRI revealed significant alterations in the PVN and SP5C regions of migraine patients (A). Detailed characterization of healthy controls and migraine patients in the fMRI study. (B) Schematic representation of bilateral PVN and SP5C localization within the human brain. (C, D) Representative fMRI activation maps from healthy controls (C) vs. migraine patients (D). (E, F) Statistical comparison of the PVN (E) and SP5C (F) regions between migraine patients and healthy controls. HC: healthy controls, MP: migraine patients. All values are presented as the mean ± SEM. ^**^ *p* < 0.01 vs. HC, two‐tailed t‐test.

The aforementioned study detailed the regulatory role and intrinsic mechanisms of the PVN^CRF^‐SP5C^Glu^ circuit in nociceptive sensitization within a mouse migraine model. However, whether this specific circuit is genuinely implicated in the pathogenesis of migraine patients remains unknown. To this end, we employed fMRI to examine whether abnormalities exist in the PVN‐SP5C circuit of migraine patients (Figure 9A,B and Table S1). The results demonstrated significant alterations in the Amplitude of Low Frequency Fluctuations (ALFF) values within the PVN and SP5C regions of both brain hemispheres in eight migraine patients compared to the control group (Figure 9C–F). This suggests that the regulatory mechanism of the PVN^CRF^‐SP5C^Glu^ circuit in migraine, identified in mice, may similarly operate in migraine patients.

## 3 Discussion

In 2021, migraine affected approximately 1.2 billion people globally [36], but the precise neurobiological mechanisms remain poorly understood. In this study, we identify and characterize a previously unrecognized excitatory descending pathway from CRF‐expressing neurons in the hypothalamic PVN to glutamatergic neurons in SP5C. Through an integrative experimental approach, we demonstrate that this PVN^CRF^‐SP5C^Glu^ circuit is engaged in an NTG‐induced model of migraine and plays a central role in trigeminal nociceptive sensitization. Under NTG‐induced migraine‐like conditions, a substantial activation of PVN^CRF^ neurons is observed, and their synaptic terminals excessively release CRF neuropeptides into the SP5C. These CRF neuropeptides activate CRFR2 receptors rather than CRFR1 receptors on SP5C^Glu^ neurons, which leads to aberrant neuronal excitation of SP5C^Glu^ neurons, ultimately resulting in migraine‐like allodynia. Moreover, clinical fMRI findings reveal structural and functional abnormalities in the PVN and SP5C regions of chronic migraine patients. This evidence further supports the critical role of this circuit in migraine pathophysiology.

Referring to pain regulation, the PVN is increasingly recognized as a functionally heterogeneous hub, comprising multiple neuropeptidergic populations that project to distinct brainstem and spinal targets [14, 21, 37, 38]. Substantial clinical and preclinical evidence has revealed that the alteration of PVN excitability contributes to the chronification and sensitization of migraine [6, 21, 39, 40, 41]. Our findings expand this framework by showing that the PVN^CRF^ neurons preferentially innervate excitatory targets within the SP5C. Given that the enhancement of SP5C^Glu^ neuron excitability is responsible for the central sensitization of trigeminal nociceptive transmission [42], we further verified that the PVN^CRF^ neurons regulate migraine‐like allodynia by innervating the SP5C^Glu^ neurons through chemogenetic inhibition of PVN^CRF^‐SP5C^Glu^ circuit. These results complement earlier work identifying an analgesic role for PVN‐derived oxytocin projections to GABAergic SP5C neurons [14], and suggest an evolutionarily adaptive dichotomy in which PVN^CRF^ neurons promote migraine by pathologically activating SP5C^Glu^ neurons, whereas PVN^OXT^ neurons suppress migraine by activating SP5C^GABA^ neurons. ACC and IC have been identified as key cortical regions involved in the pathogenesis of pain sensitization [24]. Chemogenetic inhibition of the PVN–SP5C pathway suppressed activation of both ACC and IC in the NTG‐induced migraine model, suggesting that their engagement is functionally dependent on ascending input from the PVN–SP5C circuit. However, dual retrograde tracing revealed barely overlapping populations of PVN neurons projecting to SP5C vs. those innervating ACC/IC. This anatomical segregation indicates that PVN neurons targeting SP5C do not directly regulate ACC/IC neuronal activity; instead, they modulate migraine indirectly by gating ascending nociceptive signaling from SP5C to ACC/IC.

‌In recent decades, a growing body of research has demonstrated that CRF signaling in brain regions beyond the hypothalamus (such as the prefrontal cortex, amygdala, and periaqueductal gray) plays a role in modulating pain perception and pain‐related emotional behaviors [43, 44, 45]. Acute pain stimuli can trigger CRF release from both peripheral and central neurons [46, 47, 48]. However, a comprehensive understanding of the brain mechanisms underlying CRF‐mediated migraine modulation is still lacking. With the help of a neuronal‐ and circuit‐specific CRF‐3.0 biosensor, we have achieved real‐time in vivo detection of CRF activity in PVN^CRF^‐SP5C^Glu^ circuit. We found that the NTG injection, which induced migraine‐like conditions, led to an increase in the release of the CRF neuropeptide within this circuit. As an excitatory neurotransmitter, the binding of CRF to its receptors contributes to the neuronal activation [49, 50, 51]. Indeed, the introduction of exogenous CRF protein enhanced the excitability of PVN‐innervating SP5C^Glu^ neurons during electrophysiological recording. Since our neural tracing showed that the SP5C^Glu^ neurons were primarily innervated by the CRF neurons in the PVN, these findings suggest that CRF signaling within the PVN^CRF^‐SP5C^Glu^ circuit plays a pivotal regulatory role in the central sensitization of migraine.

The CRF receptors mainly include two types: CRFR1 and CRFR2. CRFR1 is predominantly expressed in the central nervous system (e.g., pituitary gland and cerebral cortex) [31, 32], whereas CRFR2 is primarily localized to peripheral tissues including the cardiovascular system and gastrointestinal tract [33, 34]. Distinct differences exist in their tissue distribution and functional regulation [35]. CRF exhibits the highest binding affinity for CRFR1 but also binds to CRFR2 at very high concentrations. In previous pharmacological experiments, the activation of these two receptors was regarded as having counter‐regulatory action in nociception [52, 53, 54]. The prevailing model posits that pain initiation triggers CRFR1 activation, inducing marked hyperalgesia to prevent further tissue injury. This initial response is subsequently followed by sustained CRFR2 activation, which generates an analgesic effect to terminate pain. The functional dichotomy of CRF signaling thus appears to stem from the differential distribution of CRFR1 and CRFR2 across neural circuits [55]. However, our study reveals a distinct mechanism in the SP5C brainstem pain integration nucleus: pharmacological blockade of CRFR2, rather than its activation, abolishes both CRF‐induced excitation of PVN‐innervating SP5C^Glu^ neurons and recurrent NTG‐triggered cephalic cutaneous allodynia. This unexpected finding suggests that CRF receptor function exhibits neural circuit‐specific modulation. Under pathological conditions, the CRF system of SP5C demonstrates spatial heterogeneity in pain processing, contrasting sharply with the established “downstream analgesic” role of CRFR2 in limbic regions such as the amygdala.

Upon binding to receptors CRF1R/CRF2R, CRF primarily triggers G protein‐coupled signaling pathways to modulate pain [56]. Activation of Gsα stimulates adenylate cyclase, elevating cAMP levels and activating PKA. This kinase phosphorylates transcription factors like CREB, regulating gene expression (e.g., pro‐opiomelanocortin derivatives)—a core mechanism for HPA axis activation [57]. Concurrently, Gq protein activates phospholipase C, hydrolyzing PIP_2_ to generate IP_3_ and DAG, mobilizing intracellular calcium, and activating PKC to alter neuronal excitability [58]. CRF receptors also recruit β‐arrestin (regulated by GRK phosphorylation), inducing receptor desensitization and endocytosis while initiating G protein‐independent pathways (e.g., MAPK cascades). These multifaceted pathways—spanning cAMP flux, calcium dynamics, and kinase networks—collectively enable CRF to fine‐tune cellular adaptations to stressors. In migraine mouse models, the specific intracellular signaling pathways through which CRFR2 activation modulates SP5C^Glu^ neurons require further investigation.

By integrating anatomical tracing, chemogenetic manipulations, behavioral assays, and electrophysiological recordings, our study provides deep insight into the contributions of this descending PVN^CRF^‐SP5C^Glu^ circuit to migraine sensitization. Our findings also align with clinical neuroimaging data showing PVN and SP5C alterations in patients with migraine, further indicating the structural and functional link between hypothalamic regulation and brainstem pain circuits in humans.

Several limitations should be acknowledged. While the NTG model captures key aspects of migraine, it does not reproduce all features of the human condition, including aura or cortical spreading depression. In substantial preclinical studies, the NTG‐induced murine migraine model exhibited both mechanical and thermal allodynia, characterized by hypersensitivity to mechanical, heat, and cold stimuli. To avoid causing excessive and unnecessary harm to the mice, we used only cold stimuli to assess thermal allodynia. However, this does not indicate that the NTG model fails to elicit the heat allodynia. In addition, the upstream regulators of PVN^CRF^ neurons activity remain to be identified. It will be important for future studies to investigate how monoaminergic inputs, such as serotonergic or noradrenergic projections, influence CRF neuron excitability during migraine states.

In conclusion, our findings define a descending CRF‐dependent glutamatergic circuit from the PVN to the SP5C that contributes to migraine‐associated trigeminal sensitization (Figure S5). This pathway operates in functional opposition to previously reported PVN‐mediated analgesic mechanisms and represents a key interface between hypothalamic stress responses and brainstem nociceptive processing. Targeting CRFR2 signaling within this pathway may offer novel therapeutic opportunities for stress‐related migraine and other centrally sensitized pain disorders.

## 4 Materials and Methods

### 4.1 Animal

Adult male wild‐type C57BL/6J mice (8–12 weeks old, 20–25 g) were purchased from Vital River Laboratory Animal Technology (Beijing, China) Co., Ltd. Adult male OXT‐Cre and CRF‐Cre transgenic C57BL/6J mice were purchased from Cyagen Biosciences (Suzhou, China) Co., Ltd and Biocytogen Pharmaceuticals (Beijing, China) Co., Ltd, respectively. Mice were housed in groups of 2–4 per cage, with environmental conditions maintained at 22–26°C and 40%–60% humidity, and a 12‐h light/dark cycle, and the food and water of sufficient supply. All animal experiments were performed in accordance with ARRIVE Guidelines and approved by the Animal Care Committee of Qingdao University (QDU‐AEC‐2024608).

### 4.2 Chronic Migraine Model Establishment and CGRP Antibody Administration

The chronic migraine mouse model was established by repeated injection of nitric oxide donor NTG as described previously [15]. The 10 mg/1 mL of NTG stock solution was dissolved in propylene glycol (T‐021, Sigma‐Aldrich). Such a solution was freshly diluted in normal saline to achieve a final concentration of 1 mg/ml. The vehicle control was made of normal saline containing 10% propylene glycol (W294004, Sigma‐Aldrich). Mice were injected intraperitoneally with NTG (10 mg/kg) or the same volume of vehicle every other day for up to five injections. Given the large clinical evidence that anti‐CGRP drugs are beneficial in the prophylaxis of chronic migraine [59], a humanised CGRP monoclonal antibody (fremanezumab, HYP99019, MedChemExpress) was used to strengthen and validate the translational value of the present model of NTG‐evoked hypersensitivity. The 30 mg/kg of fremanezumab (i.p.) significantly alleviated headache in mice, which exhibited a half‐life of 52.4 h in the mouse model [18]. Based on the pharmacokinetic profile, we administered this dosage of fremanezumab or an IgG isotype control antibody (conAb, HYP80757, MedChemExpress) on days 0, 4, and 8 during chronic NTG treatment, ensuring sustained plasma concentrations throughout the experimental window.

### 4.3 Behavioral Assessments

Behavioral assessments were conducted at 2 h before and at 1, 2, and 4 h after the first NTG injection on day 1, at 2 h before the second to fifth NTG injections on day 3, day 5, day 7, and day 9, respectively, and 12:00 on day 10. Mice were placed in a quiet, dim (20 lux) room for 30 min prior to behavioral tests to allow them to habituate to the testing environment. Given that persistent cutaneous allodynia is a hallmark of migraine chronification [60], we assessed the mechanical and cold allodynia using von Frey filaments and acetone assays, respectively, focusing on the trigeminal nerve‐innervated periorbital region.

### 4.4 Von Frey Test

Mechanical allodynia was assessed by quantifying the periorbital mechanical threshold in response to von Frey filaments (ranging from 0.04 to 2 g; Stoelting, IL, USA) using the up‐and‐down method [61, 62]. Mice were acclimatized to handling and innocuous mechanical stimuli for 10 min daily for at least 5 consecutive days prior to periorbital mechanical threshold measurements. During the von Frey test, the filaments were applied perpendicularly to the periorbital skin with ascending intensity, starting at 0.16 g, until the filaments gently bent into a “C” shape. The filament was held in place for 3 s, and a positive response was defined as a rapid head withdrawal, scratching, or tremor. The von Frey filament that elicited a positive response was recorded. Three repeated trials were performed with a 5‐min interval between each trial, and the average value across the three trials was taken as the periorbital mechanical threshold.

### 4.5 Acetone Test

To assess cold allodynia, a drop of acetone was applied to the periorbital region of the mouse, and the positive responses, including head scratching or shaking, were observed. In brief, the mice were placed in a transparent plexiglas chamber with a metal mesh floor in front of a high‐resolution camera (FDR‐AX40, SONY, Japan). A drop of 20 µL acetone was applied to the periorbital region of the head using a syringe. During the 1‐min observation period following acetone application, the duration of positive responses was recorded to evaluate cold allodynia.

### 4.6 Stereotaxic Surgery and Viral Injections

For stereotaxic surgery, mice were anesthetized with 2%–4% isoflurane and placed in a stereotaxic apparatus (RWD Life Science, Shenzhen, China). An erythromycin ophthalmic ointment was used to prevent the cornea from drying out, and a heating pad was applied to maintain body temperature. Viruses were injected using pulled glass microelectrodes (GC‐3.5, RWD Life Science, Shenzhen, China) connected to a 10‐microliter syringe into one of the following target regions according to the experiment: PVN (AP −0.7 mm, ML ±0.3 mm, DV −4.8 mm), SP5C (AP −6.5 mm, ML ± 1.7 mm, DV −3.9 mm), IC (AP +1.7 mm, ML ±3.0 mm, DV −2.7 mm) and ACC (AP +0.7 mm, ML ±0.3 mm, DV −1.8 mm), with AP, ML, and DV representing the anteroposterior, mediolateral, and dorsoventral distance (mm) from bregma, respectively. The coordinates were determined using Paxinos and Watson's “The Mouse Brain in Stereotaxic Coordinates.” The micropipette was left in place for 5 min after injection to ensure complete delivery and minimize diffusion.

To investigate the activation of PVN ^CRF^ neurons and PVN ^OXT^ neurons after repeated NTG injection, we unilaterally injected AAV2/9‐DIO‐EGFP (titer: 3 × 10^12^ vg/mL, OBiO, Shanghai, China) into the PVN of CRF‐Cre and OXT‐Cre mice, respectively, followed by 3 week's recovery and repeated NTG injection. The mice were sacrificed for c‐Fos immunofluorescence staining 2 h after the last NTG injection.

For anterograde monosynaptic tracing experiments, an HSV‐based anterograde monosynaptic tracer system [23] was used to map the PVN^CRF^ neuron‐innervating neurons in the SP5C. The HSV‐based anterograde monosynaptic tracer system, which consists of a modified HSV‐tracer (Hs06) and an amplification helper, was purchased from BrainCase Co., Ltd. (Wuhan, China). First, AAV2/9‐UL26.5p‐DIO‐cmgD (titer: 1.33 × 10^12^ vg/mL, BrainCase) and AAV2/9‐hSyn‐DIO‐EGFP‐T2A‐Her2CT9 (titer: 1 × 10^12^ vg/mL, BrainCase) were mixed as the amplification helper and injected into the PVN of CRF‐Cre mice. Two weeks later, the Hs06 (H129ΔgD‐hUbC‐mCherry‐P2A‐scHer2::gd, titer: 3 × 10^8^ plaque‐forming units (PFU)/mL) was injected into the same injection site. Five days after the Hs06 injection, the mice were perfused to observe the mCherry‐expressing neurons in the SP5C.

For the monosynaptic retrograde tracing experiments, AAV2/Retro‐hSyn‐mCherry (titer: 2.80 × 10^12^ vg/mL, OBiO) or AAV2/Retro‐hSyn‐EGFP (titer: 2.16 × 10^12^ vg/mL, OBiO) was injected into the ACC, IC, or SP5C.

For chemogenetic manipulation of PVN^CRF^ neurons, AAV2/9‐DIO‐hM4Di‐mCherry (titer: 5.04×10^12^ vg/mL, OBiO) or AAV2/9‐DIO‐hM3Dq‐mCherry (titer: 5.02 × 10^12^ vg/mL, OBiO) was bilaterally injected into the PVN of CRF‐Cre mice. For chemogenetic manipulation of the SP5C‐projecting PVN^CRF^ neurons, a retrograde tracing AAV vector encoding Flp recombinant (AAV2/Retro‐DIO‐FLP‐EGFP, titer: 2.16 × 10^13^ vg/mL, OBiO) was bilaterally injected into the SP5C, and AAV2/9‐DIO‐fhM4Di‐mCherry (titer: 5.04 × 10^12^ vg/mL, OBiO) was bilaterally injected into the PVN. For chemogenetic manipulation of the PVN‐innervating SP5C glutamatergic neurons, an anterograde transsynaptic AAV vector encoding Cre recombinant (AAV2/1‐hSyn‐EGFP‐Cre, titer: 1.67 × 10^13^ vg/mL, OBiO) was bilaterally injected into PVN, and AAV2/9‐CaMKII‐DIO‐hM4Di‐mCherry (titer: 3.0 × 10^12^ vg/mL, OBiO) was bilaterally injected into the SP5C. At least 28 days after the viral injections, the mice were intraperitoneally injected with clozapine‐N‐oxide (CNO) (2 mg/kg) or its vehicle (1% DMSO in normal saline) and allowed to rest for 30 min before behavioral tests.

For real‐time measurement of PVN^CRF^ neuron activity, an AAV vector encoding a fluorescent calcium indicator (AAV2/9‐DIO‐GCaMP6m, titer: 1.00 × 10^12^ vg/mL, OBiO) was unilaterally injected into the PVN of CRF‐Cre mice. Two weeks later, an optical fiber (Φ1.25 mm, 200/0.37, Inper, Hangzhou, China) was implanted 0.5 mm above the PVN.

For in vivo recording of CRF peptide release from SP5C projections of PVN neurons, AAV2/1‐hSyn‐EGFP‐Cre (titer: 1.67 × 10^1^ ^3^ vg/mL, OBiO) was unilaterally injected into the PVN, while an AAV vector encoding the G‐protein coupled receptor activation‐based CRF (GRAB CRF3.0) biosensor (AAV2/9‐CaMKII‐DIO‐CRF3.0‐GFP, 1.00×10^1^ ^2^ vg/mL, OBiO) was injected into the ipsilateral SP5C. Two weeks later, an optical fiber was implanted into the SP5C.

### 4.7 In Vivo Fiber Photometry Recording

We used a fiber photometry system to collect calcium and CRF signals. Fluorescent signals of calcium and CRF were recorded by stimulating neurons expressing GCaMP6m and CRF3.0 biosensors with a 488‐nm LED (20–30 µW at the fiber tip), while isosbestic control signals were obtained by stimulating the same neurons with a 405‐nm LED (20–30 µW at the fiber tip). To examine the responses of PVN^CRF^ neurons and CRF release after NTG injection, dynamic changes in calcium and CRF biosensor fluorescent signals were recorded 30 min after NTG administration, and fluorescent signals 5 min before NTG administration were recorded as baseline. To assess evoked changes in the activity of PVN^CRF^ neurons, we recorded short‐term calcium signals during cutaneous allodynia evoked by an innocuous Von Frey filament (0.07 g). The dynamic changes in PVN^CRF^ neurons’ activity and CRF release were determined by the fluorescence signal change ratio (ΔF/F0, where ΔF = Ft–F0), which was calculated from the raw data generated by the fiber photometry recording system (THINKERTECH, Nanjing, China). The ΔF/F% values were calculated as (Ft–F0)/F0 × 100%, where F0 represents the averaged baseline fluorescent signal recorded either 300 s before NTG injection or 5 s before the Von Frey filament stimulus, and Ft denotes the simultaneous fluorescence signal captured either 1800 s post‐NTG injection or 10 s after the Von Frey filament stimulus. The exported data underwent statistical analysis and were presented as average plots and heatmaps. The area under the curve (AUC) of ΔF/F% values was analyzed as the sum of fluorescence changes over 1800 s after NTG injection to investigate the changes in PVN^CRF^ neurons’ activity and CRF release. The peak ΔF/F% values after the Von Frey filament stimulus were calculated to evaluate the activities of PVN^CRF^ neurons in response to the transient cutaneous allodynia.

### 4.8 Tissue Processing and Immunohistochemistry

Mice were deeply anesthetized with 1% sodium pentobarbital (50 mg/kg) and perfused intracardially with 4% paraformaldehyde (PFA) in 0.01 M phosphate buffer solution (PBS). Next, the brain samples were obtained and then successively immersed in 4% paraformaldehyde in 0.01 M PBS at 4°C for 12 h and 30% sucrose in 0.1 M PBS at 4°C for 12 h. After the sample preparation, brains were embedded in optimal cutting temperature (OCT) compound and cut into 30 µm sections using a cryostat microtome (CM860 UV, Leica, Germany). The prepared brain sections were permeabilized with 0.5% Triton X‐100 for 15 min at room temperature (RT), and then blocked with 10% donkey serum for 1 h at RT. For immunofluorescent staining, the sections were incubated with c‐Fos (rabbit, 1:3000, Cell Signaling Technology, 2250), TRPV1 (rabbit, 1:500, Abcam, ab6166), Vglut2 (mouse,1:200, Abcam, ab211869), CRFR1 (goat, 1:500, Thermofisher Scientific, PA5‐18801), and CRFR2 (rabbit, 1:500, Thermofisher Scientific, PA5‐23129) primary antibodies for 12 h at 4°C, followed by the donkey Alexa Fluor 488 or 568 secondary antibodies (1:1000, Abcam, ab150073, ab175470, ab150105, ab175704) for 2 h at RT and with 4,6‐diamino‐2‐phenyl indole (DAPI) (P0131, Beyotime Biotechnology, Shanghai, China).

To validate the Cre activities in CRF‐Cre or OXT‐Cre transgenic mice and identify the labeled neuronal type, we performed a combined RNAscope in situ hybridization with immunostaining, following the protocol described in previous literature [63]. The CRF (C1‐316091), OXT (C2‐493171), Fos (C4‐316921), Slc17a6 (C3‐319171), and Slc32a1 (C2‐319191) RNA hybridization antisense probes were purchased from Advanced Cell Diagnostics Co., Ltd (CA, USA) and used to detect the mRNA expression of CRF, OXT, c‐Fos, vesicular glutamate transporter 2 (Vlut2, glutamatergic neuron marker) and vesicular GABA transporter (Vgat, GABAergic neuron marker), respectively. In situ hybridization was performed on frozen sections using the RNAscope Fluorescent Multiplex Kit (Advanced Cell Diagnostics, CA, USA, 323100) following the manufacturer's protocol. After completing the RNAscope staining, the sections were then blocked for the immunofluorescence staining and mounted with DAPI. The primary antibodies used for combined staining were: mCherry (rabbit, 1:200, Cell Signaling Technology, 43590), EGFP (rabbit, 1:200, Rockland, 600‐401‐215). The secondary antibodies used were donkey Alexa Fluor 488 or 568 (1:1000, Abcam, ab150073, ab175470).

Tissue images were captured using the VS120 slide scanner system (Olympus, Tokyo, Japan) or FV3000 confocal microscope (Olympus, Tokyo, Japan). The pictures were input into NIH ImageJ software for analysis, with 4 continuous sections per animal (90‐um interval). Specifically, the threshold was controlled by identical parameters to standardize the detection of the target signals. The expression of c‐Fos in CRF+ and OXT+ PVN neurons was detected to evaluate the activation of PVN^CRF^ neurons and PVN ^OXT^ neurons. The validation of the Cre activities in the CRF‐Cre or OXT‐Cre transgenic mice was determined by detecting the expression of CRF and OXT in Cre‐labeled neurons. The proportions of CRF neurons and OXT neurons in the SP5C‐projecting PVN neurons were determined by calculating the expression of CRF and OXT in EGFP‐labeled neurons. The neuronal type of PVN‐innervating SP5C neurons was identified by calculating the percentages of Slc17a6+ and Slc32a1+ neurons in the mCherry‐labeled SP5C neurons. The expression of CRFR1 and CRFR2 in the glutamatergic neurons of SP5C was detected by calculating the co‐localization of CRFR1 and CRFR2 with Vglut2.

### 4.9 Cannula Implantation and Drug Administrations

To antagonize CRF receptors in the SP5C, the customized drug delivery cannula (O.D.0.41×I.D.0.25 mm, RWD Life Science, Shenzhen, China) was implanted into bilateral SP5C and cemented on the skull.

For in vivo pharmacological inhibition of the CRFR1 or CRFR2 in SP5C, we selected NBI35965 (1 µmol/L, MedChemExpress, HY‐162872) and antisauvagine‐30 (1 µmol/L, MedChemExpress, HY‐P1107) to antagonize the CRFR1 and CRFR2, respectively, with 300 nl per side. The concentration and volume of the drugs were determined based on the previous studies [64]. The drugs were infused using a perfusion pump (R462, RWD Life Science, Shenzhen, China) at a rate of 50nL/min. Following the drug delivery, the injection cannula was kept in place for an additional 5 min to prevent the drug backflow.

### 4.10 Electrophysiology

Four weeks after the viral injection, electrophysiology was performed to investigate the effects of CRF, CRFR1, and CRFR2 on the activity of PVN‐innervating SP5C glutamatergic neurons. Four weeks after the viral injection, mice were anesthetized with 4% isoflurane and intracardially perfused with cold, oxygenated section artificial cerebrospinal fluid (sACSF), which contained the following concentrations (in mM): NaCl 120, KCl 2.5, NaH2PO4 1.0, NaHCO3 26.0, CaCl2 2.5, MgSO4 1.3, and D‐glucose 1.0. The acute coronal slices (300 µm) containing the SP5C region were sectioned using a Leica vibrating microtome (VT1200s, Leica, Germany), then recovered in oxygenated incubation‐ACSF (in mM): KCl 2.5, NaH2PO4 1.25, NaHCO3 25, D‐glucose 25, CaCl2 0.5, MgSO4 7, Sodium Pyruvate 3.1, Cloline choloride 110, Sodium Ascorbate 11.6 for 1 h at 31°C and subsequent 1 h at room temperature. After recovery, the SP5C neurons were selected using the differential interference contrast optics, a microscope (BX51WI, Olympus, Japan), and a CCD camera (EXi Aqua, QImaging, Surrey, BC, Canada). Whole‐cell patch‐clamp recordings were performed in gap‐free acquisition mode, using pClamp 10 software (Molecular Devices, Sunnyvale, CA) and Multiclamp 700B amplifier. The pulled patch pipettes (3–5 MΩ) were filled with patch pipette solution (in mM): K‐gluconate 145, NaCl 5, MgCl2 1, EGTA 0.2, HEPES 10, ATP‐Mg^2+^ 2, GTP‐Na^+^ 0.1. Selected neurons with a resting membrane potential ≤ −60 mV, series resistance ≤ 30 MΩ, and a change in these parameters ≤ 20% were recorded, and their spontaneous action potential firing rates were detected by injecting depolarizing current steps (15 to 115 pA, 1 s). To investigate the effects of CRF, CRFR1, and CRFR2 on the activity of PVN‐innervating SP5C glutamatergic neurons, the drugs were added to the ACSF from their stock solutions to obtain known concentrations in the superfusate: CRF 200 nmol/L [65], Antalarmin (CRFR1 antagonist) 1 µmol/L [66], antisauvagine‐30 1 µmol/L [51]. To examine whether CRF can modulate the peripheral nociceptive inputs, we recorded the changes in optically‐evoked excitatory postsynaptic currents (oEPSCs) of the TRPV1‐expressing primary afferent‐innervating SP5C neurons following CRF (200 nmol/L) application. The TRPV1‐expressing primary afferents labeled with ChR2 were stimulated by flashing 470‐nm light. Brief pulses of light (1–3 ms duration, 2 mW/mm^2^) were delivered by an LED (Prizmatix) through the 40× water‐immersion objective under the control of acquisition software.

### 4.11 fMRI Experimental Methods

Adults with migraine, as defined by the International Headache Society criteria (ICHD‐3) [67], and the healthy controls were recruited from the affiliated hospital of Zhengzhou University. All participants signed the informed consent. The human study was approved by the Henan Provincial People's Hospital [approval number: Ethics (2021) No. (146)] and adhered to the Declaration of Helsinki and all applicable ethical guidelines.

### 4.12 Inclusion and Exclusion Criteria

Migraine patient inclusion criteria:

1. Meeting the diagnostic criteria for migraine according to the International Classification of Headache Disorders, third edition (ICHD‐3);
2. Age ≥18 years;
3. Informed consent was obtained from patients and their families.

Healthy control group inclusion criteria:

1. Age‐, sex‐, and education level‐matched healthy individuals;
2. No history of migraine or other chronic pain disorders, and no family history of headache;
3. Informed consent obtained from participants or their families.

Exclusion criteria for all subjects:

1. Patients with other types of headaches (e.g., secondary headaches caused by encephalitis, meningitis, vascular diseases, or intracranial hypertension);
2. Exclusion of somatic symptoms attributed to depression or anxiety;
3. History of alcohol/tobacco abuse or preventive medication use within 1 year;
4. Contraindications for MRI (e.g., cardiac pacemakers, metal implants, claustrophobia);
5. History of organic brain diseases;
6. Female participants who were pregnant or menstruating.

### 4.13 Imaging Data Acquisition

Scans were performed using a MAGNETOM Prisma 3T MR scanner (Siemens Healthineers, Forchheim, Germany) with a standard 64‐channel head coil. Participants lay supine on the examination table, wearing earplugs to reduce scanner noise, and foam pads were used to minimize head motion. They were instructed to remain still, keep their eyes closed (without falling asleep), and avoid focused thinking.

### 4.14 Structural Imaging

A 3D T1‐weighted magnetization‐prepared rapid gradient‐echo (MPRAGE) sequence was used with the following parameters: repetition time (TR) = 2300 ms, echo time (TE) = 2.28 ms, slice thickness = 1 mm, no gap, 192 slices, field of view (FOV) = 260 × 260 mm^2^, matrix size = 256 × 256, flip angle = 8°, and voxel size = 1 × 1 × 1 mm^3^.

### 4.15 Functional Imaging

An echo‐planar imaging (EPI) sequence was employed with parameters: TR = 2000 ms, TE = 35 ms, slice thickness = 2.2 mm, no gap, 75 slices, FOV = 207 × 207 mm^2^, matrix size = 94 × 94, flip angle = 80°, and voxel size = 2.2 × 2.2 × 2.2 mm^3^.

### 4.16 Statistical Analysis

Data were presented as mean ± SEM and analyzed using the Statistical Package for the Social Sciences software (version 17.0; SPSS Inc., Chicago, IL, USA). Normality and homogeneity of variance were assessed using the Shapiro‐Wilk test and Brown‐Forsythe test, respectively. Normally distributed data with homogeneous variance were analyzed using the unpaired or paired t tests, one‐way, two‐way, or repeated measurement ANOVA followed by Bonferroni's post hoc tests. Other data sets were analyzed with a nonparametric test. The level of statistical significance was set at *p *< 0.05.

## Author Contributions

J.B., X.W., L.Y., W.W., X.L., Y.G., Z.X., N.T., and L.C. performed research; M.M., X, J., X.X., and J.B. analyzed data; X.Z., X. J., X.X., and M.W. conceived the project and wrote the manuscript.

## Conflicts of Interest

The authors declare no conflicts of interest.

## Supporting information




**Supporting File**: advs74608‐sup‐0001‐SuppMat.docx.

## Data Availability

The data that support the findings of this study are available from the corresponding author upon reasonable request.
