# Supplementary material for: PVNCRF Neurons Regulate Migraine‐Like Allodynia by Activating CRFR2 on Spinal Trigeminal Caudalis Glutamatergic Neurons
Source: Adv Sci (Weinh). 2026 Feb 28;13(29):e20530. doi: 10.1002/advs.202520530 (PMC13205600; doi:10.1002/advs.202520530)
Supplement: Supplementary file 1 — Supporting File: advs74608‐sup‐0001‐SuppMat.docx. [file ADVS-13-e20530-s001.docx]

**Fig. S1: Repeated NTG injections activate PVN^CRF^ neurons**

**A.** Experimental schedule for viral injections, repeated NTG injections and c-Fos staining. **B.** Validating the specificities of CRF-Cre and OXT-Cre lines and the accuracy of viral injections. Scale bars, 100 μm. **C.** Representative images and graphs showing the overlap between PVN^OXT^ neurons and c-Fos, and between PVN^CRF^ neurons and c-Fos, respectively. White arrows indicate merged neurons. Scale bars, 100 μm. All values are presented as the mean ± SEM. ** *p*<0.01 vs. Vehicle, two tailed *t* test.

**Fig. S2: CNO administration has no effects on periorbital mechanical and thermal sensation of mice injected with the mCherry-control virus**

**A.** Experimental schedule for viral injections into the PVN, NTG and CNO injections and behavioral tests. **B.** Frontal sections showing mCherry-expressing PVN^CRF^ neurons in the PVN. **C, D.** Validating the specificity of the CRF-Cre line and accuracy of viral injections. Scale bars, 100 μm. **E, F.** Von Frey (**E**) and acetone (**F**) tests showing effects of CNO administration on periorbital mechanical and thermal sensation in CRF-Cre male injected mCherry-control virus into the PVN. All values are presented as the mean ± SEM. ** *p*<0.01 vs. Vehicle+DMSO, Bonferroni *post-hoc* test after two-way ANOVA (**E:** NTG, F_(1,14)_ =147.603, *p* < 0.001; CNO, F_(1,14)_=0.327, *p*=0.557; interaction, F_(1,14)_=0.593, *p*=0.454; **F:** NTG, F_(1,14)_=226.941, *p*<0.001; CNO, F_(1,14)_ =0.142, *p*=0.742; interaction, F_(1,14)_=0.142, *p*=0.712).

**Fig. S3: Repeated NTG injections activate SP5C neurons, SP5C-projecting PVN neurons primarily express CRF**

**A, B.** Representative images and graphs of c-Fos-positive neurons in the SP5C of mice with repeated NTG or vehicle injections**.** Scale bars, 200 μm (left) and 50 μm (right). **C.** Experimental schedule for viral injections into the SP5C. **D.** Frontal sections showing EGFP-labelled neurons in the SP5C. Scale bars, 500 μm. **E, F.** Representative images and graphs of the overlap between SP5C-projecting PVN neurons (PVN-SP5C PNs) and CRF mRNA or OXT mRNA. Scale bars, 100 μm. All values are presented as the mean ± SEM. ** *p*<0.01 vs. Vehicle or CRF, two tailed *t* test.

**Fig. S4. Inhibition of SP5C-projecting PVN^CRF^ neurons reduces NTG-induced IC/ACC activation, and SP5C-projecting PVN neurons barely overlap with IC-/ACC-projecting PVN neurons**

**A.** Experimental schedule for viral injections into the PVN and SP5C. **B.** Frontal sections showing EGFP-expressing neurons in the SP5C and mCherry-expressing neurons in the PVN. Scale bars, 200 μm. **C, D.** Representative images and graphs showing the effects of inhibiting SP5C-projecting PVN^CRF^ neurons on the activities of the IC (**C**) and ACC (**D**) in the mice with repeated NTG or vehicle injections. Scale bars, 200 μm(left) and 100 μm (right). **E, H.** Experimental schedule for viral injections into the IC (**E**), ACC (**H**) and SP5C (**E, H**). **F, I.** Frontal sections showing EGFP-expressing projections in the SP5C (**F, I**), mCherry-expressing projections in the IC (**F**) and ACC (**I**), and distributions of neurons labeled with retrogradely transported EGFP and mCherry in the PVN. Scale bars, 200 μm (left) and 100 μm (right). **G, J.** Statistical graphs of overlapping proportions. PNs, projecting neurons. All values are presented as the mean ± SEM. ** *p*<0.01 vs. Vehicle+DMSO, **^##^** *p*<0.01 vs. NTG+DMSO, Bonferroni *post-hoc* test after two-way ANOVA (**C:** NTG, F_(1,10)_ =188.477, *p* < 0.001; CNO, F_(1,10)_ =9.327, *p* = 0.012; interaction, F_(1,10)_ = 7.321, *p* = 0.022; **D:** NTG, F_(1,10)_ =233.084, *p* < 0.001; CNO, F_(1,10)_ =29.070, *p* < 0.001; interaction, F_(1,10)_ = 29.782, *p* < 0.001).

**Fig. S5: Diagram of PVN^CRF^-SP5C^Glu^ circuit modulating NTG-induced migraine-like hypersensitivity**

Repeated NTG injection pathologically activates PVN^CRF^ neurons, leading to excessive release of CRF neuropeptides into the downstream nucleus SP5C. The elevated CRF neuropeptides aberrantly activate CRFR2 receptors on SP5C^Glu^ neurons, resulting in hyperexcitability of these neurons and ultimately triggering migraine-like hyperalgesia. PVN, hypothalamic paraventricular nucleus; CRF, corticotropin-releasing factor; SP5C, spinal trigeminal nucleus caudalis; CRFR2, corticotropin-releasing factor receptor type 2; Glu, glutamatergic.


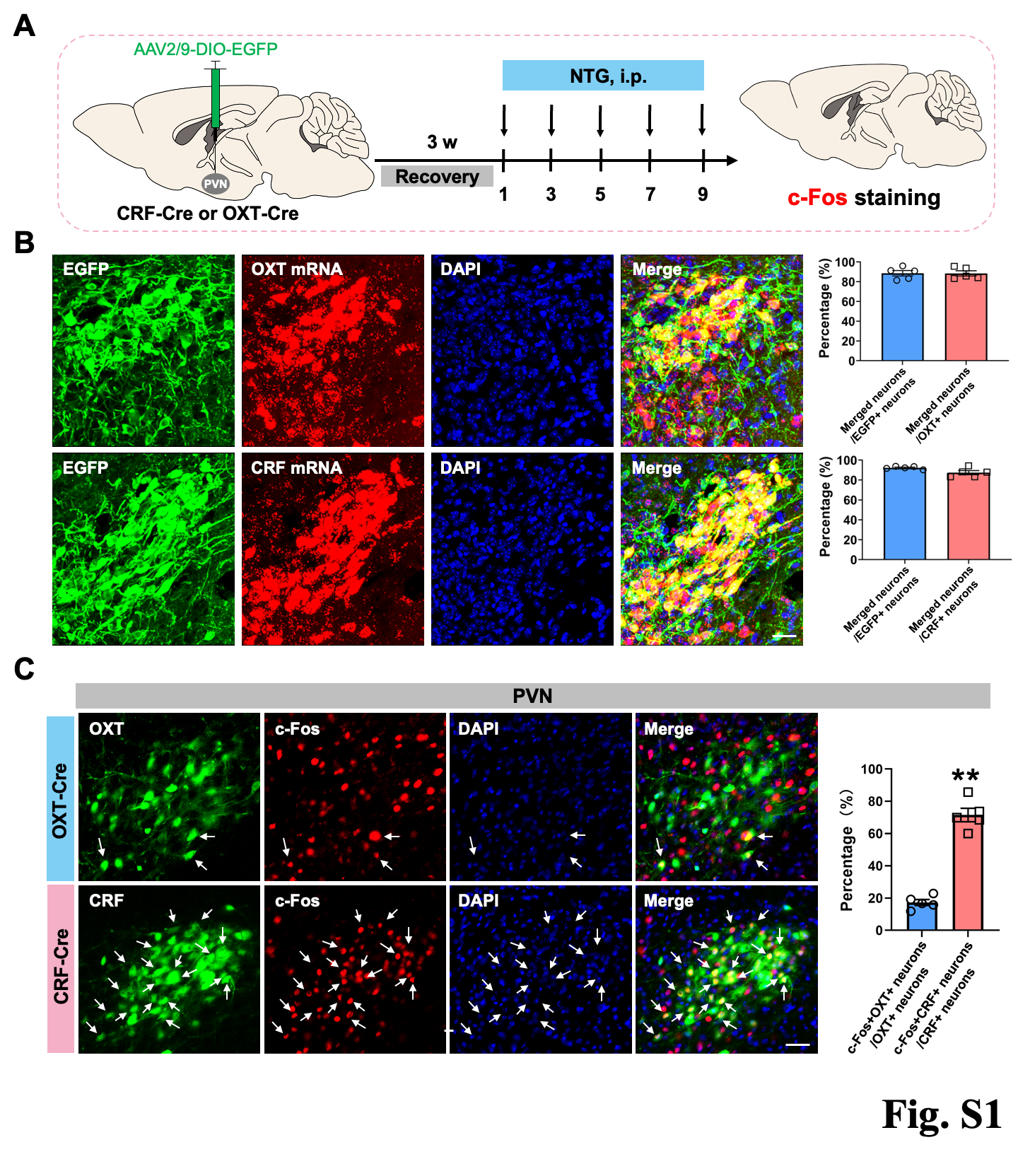

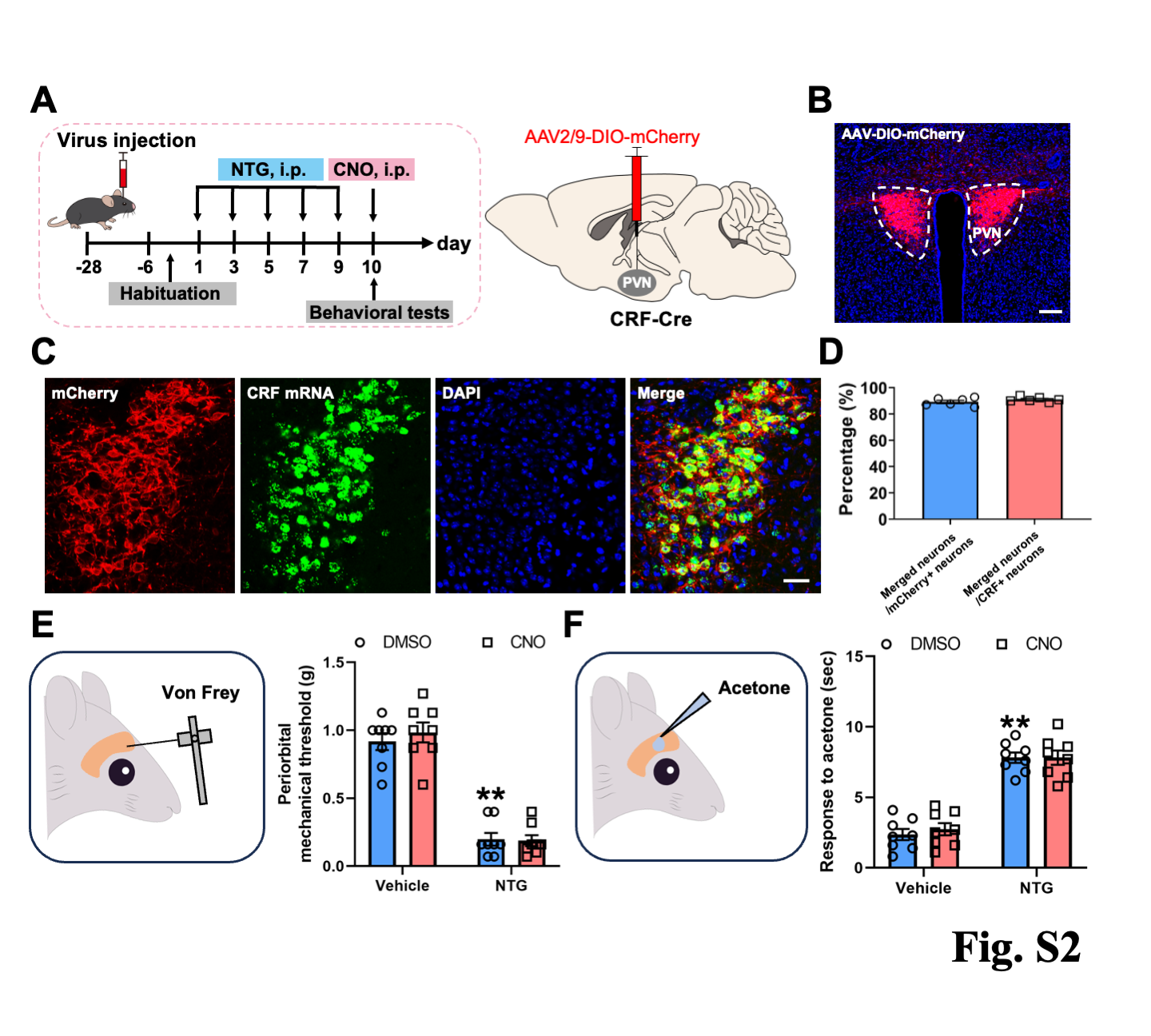

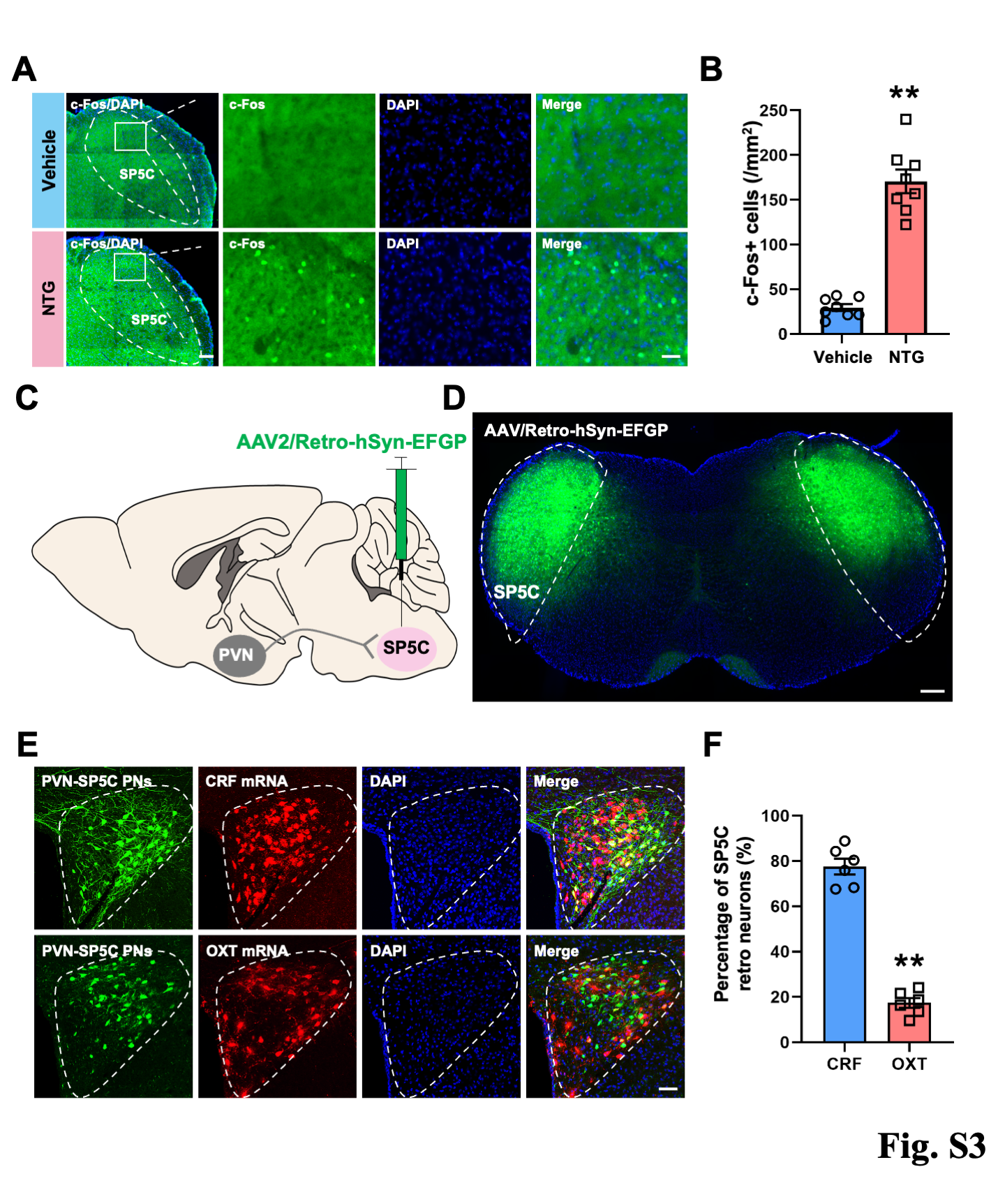

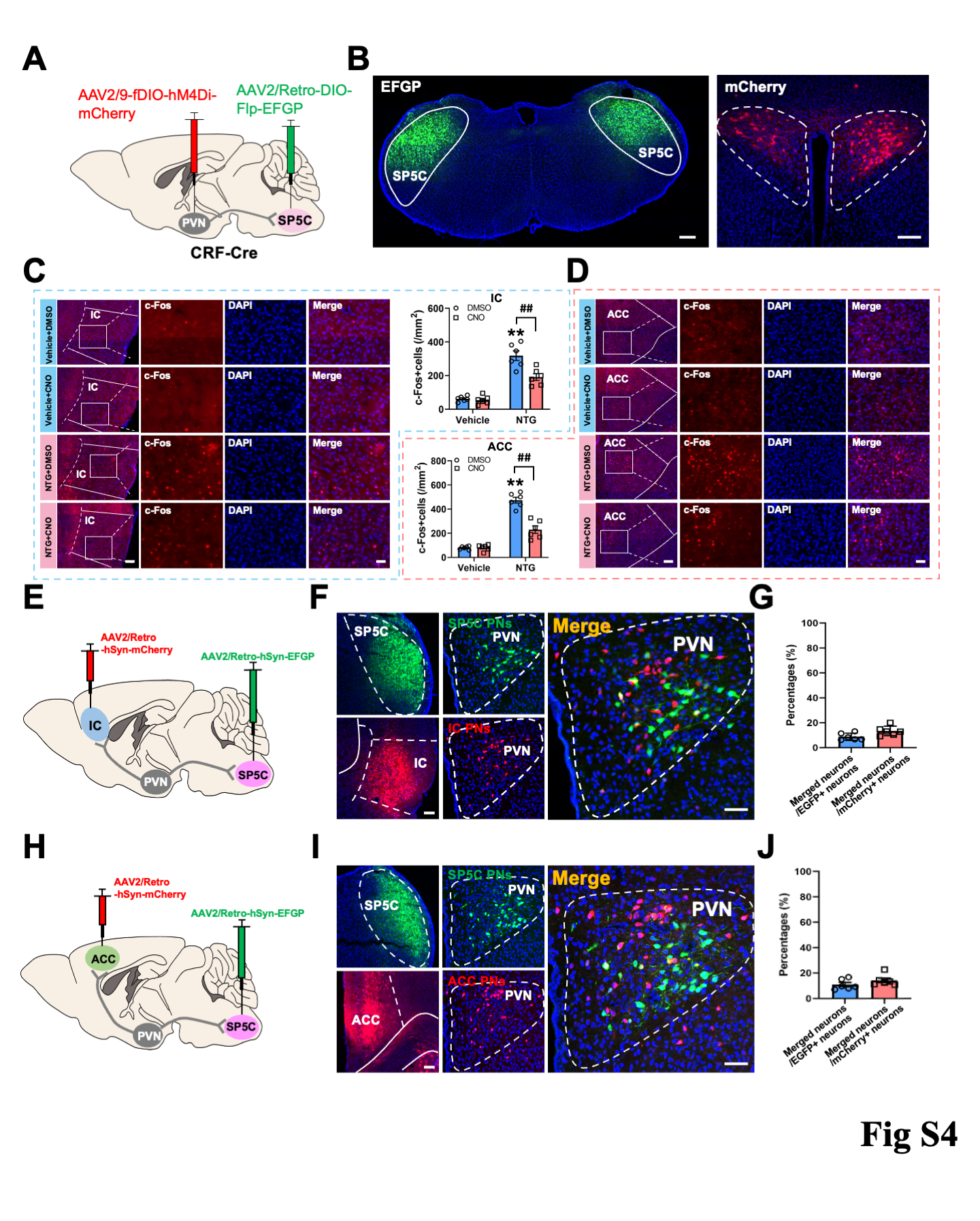

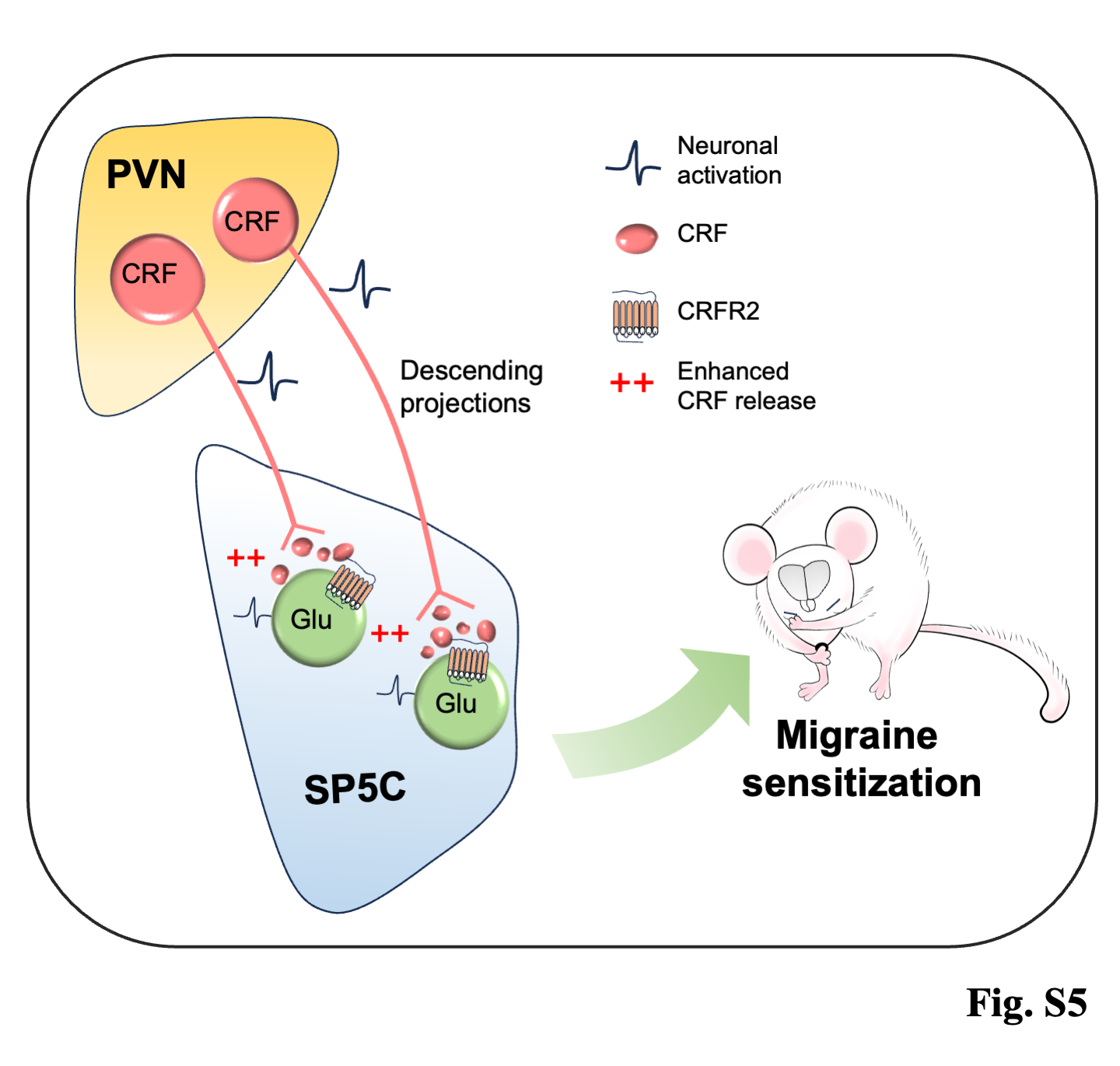


| No. | Gender | Age | Course of Disease | Duration | Frequency of Attacks | Headache Severity | Onset Time |
| --- | --- | --- | --- | --- | --- | --- | --- |
| 1 | Female | 28 | Year and a half | 2-3 hours | Once a month | Moderate | Evening (after overwork) |
| 2 | Male | 30 | More than a month | 1 hour | Once a month | Severe | None |
| 3 | Female | 26 | Fifteen years | 6-8 hours | 2-3 times a week | Moderate | After 2 p.m. generally |
| 4 | Female | 26 | Ten years | 1 hour | 1-2 times a month | Moderate | During the day at work |
| 5 | Male | 30 | Three weeks | 0.5-1 hour | Once a week on average | Moderate | After overwork |
| 6 | Female | 27 | Ten years | 2-3 hours | Once a month | Moderate | None |
| 7 | Female | 25 | Four years | 1 hour | 2-3 times a year | Moderate | During the day at work |
| 8 | Male | 35 | Three years | 1 hour | Once every six months | Mild | During the day at work |

Table S1: Record of Clinical Onset Characteristics in Migraine Patients
